# Beyond Area Under the Receiver Operating Characteristic Curve: Evaluating Predictive Performance Metrics Under Class Imbalance in Real-World Clinical Data

**DOI:** 10.2196/86379

**Published:** 2026-06-24

**Authors:** Vanessa das Graças José Ventura, Claudio Moisés Valiense de Andrade, Jussara Marques de Almeida, Bruno Porto Pessoa, Carísi Anne Polanczyk, Guilherme Fonseca do Nascimento, Eric Boersma, Heloisa Reniers Vianna, Katia de Paula Farah, Leonardo Chaves Dutra da Rocha, Marcos André Gonçalves, Milena Soriano Marcolino

**Affiliations:** 1 Medical School and University Hospital, Universidade Federal de Minas Gerais, Avenida Alfredo Balena, 110, Belo Horizonte, 30130-100, Brazil, 55 31991314221; 2 Department of Computer Science, Universidade Federal de Minas Gerais, Belo Horizonte, Brazil; 3 Hospital Júlia Kubitschek, Belo Horizonte, Brazil; 4 Department of Medicine Internal, Medical School, Universidade Federal do Rio Grande do Sul, Porto Alegre, Brazil; 5 Institute for Health Assessment and Translation for Chronic and Neglected Diseases of High RElevance (IATS-CARE), Belo Horizonte, Brazil; 6 Hospital Moinhos de Vento, Porto Alegre, Brazil; 7 Erasmus University Medical Center, Rotterdam, The Netherlands; 8 Hospital Universitário Ciências Médicas, Belo Horizonte, Brazil; 9 Department of Internal Medicine, Medical School, Medical School and University Hospital, Universidade Federal de Minas Gerais, Belo Horizonte, Brazil; 10 Telehealth Center, University Hospital, Universidade Federal de Minas Gerais, Belo Horizonte, Brazil

**Keywords:** predictive model, artificial intelligence, learning curve, area under the receiver operating characteristic curve, *F*_1_-score, performance metrics

## Abstract

**Background:**

Predictive models increasingly support clinical decision-making, although imbalanced outcome distributions are common in health care datasets and can distort performance evaluation. The area under the receiver operating characteristic curve (AUROC) remains the most frequently reported metric, despite its limited ability to reflect clinically meaningful performance under class imbalance.

**Objective:**

This study aimed to examine the influences of metric selection on the clinical interpretation of predictive models in imbalanced real-world health care data.

**Methods:**

This was a retrospective cohort study, including 17,018 hospitalized patients with COVID-19. Two predictive models using extreme gradient boosting (XGBoost) were developed to predict kidney replacement therapy (KRT) and mortality. Model performance was assessed using AUROC, macro-*F*_1_-score, class-specific precision and recall, calibration (curve, slope, and intercept), decision curve analysis, and learning curves. Standard rebalancing strategies were applied exclusively to the training data to evaluate their impact on performance.

**Results:**

KRT occurred in 9.5%, and mortality in 18.0%. Although AUROC values were high (0.928 for KRT and 0.945 for mortality), performance in the minority class was substantially lower. For KRT, precision was 0.539 and recall 0.372; for mortality, precision was 0.725 and recall 0.718. Rebalancing strategies were associated with higher recall for the minority class, but this gain was accompanied by a reduction in precision, with minimal impact on AUROC values. As a result, AUROC remained high despite clinically relevant changes in error distribution between false positives and false negatives. The learning curves show a plateau-like shape, with stable validation performance across all training set sizes for both outcomes.

**Conclusions:**

AUROC alone is insufficient to evaluate prediction models in imbalanced health care scenarios, even with rebalancing. Routine reporting of class-aware metrics, alongside learning curve analysis, is essential to support robust and clinically meaningful evaluation of predictive models, rather than their direct translation into practice.

## Introduction

Clinical prediction models are increasingly used in health care to support diagnostic, prognostic, and therapeutic decisions [1,2]. Their adoption has expanded with advances in machine learning (ML) and access to large-scale electronic health data, enabling the development of models with the potential to improve risk stratification and personalized care [3]. However, the evaluation of these models frequently relies on metrics that may not reflect real-world clinical usefulness, especially when outcome distributions are imbalanced [4].

The area under the receiver operating characteristic curve (AUROC) is the most commonly reported metric in clinical prediction research [5,6]. Although AUROC is intuitive and threshold-independent, it often overestimates performance in datasets in which one class (eg, survival or absence of disease) predominates, a common characteristic in clinical datasets [7-9]. In such settings, AUROC may suggest high discriminative ability while concealing poor sensitivity for minority-class outcomes, such as death or the need for critical interventions [7-9].

For example, risk prediction scores have long been recommended in clinical practice to guide preventive strategies, particularly in cardiovascular disease. The Framingham score, once widely used for predicting 10-year cardiovascular outcomes, illustrates the risk of relying on AUROC-based metrics [10]. Although it showed acceptable discrimination (C-statistic: 0.763 for men and 0.793 for women) [10,11], the dataset exhibited class imbalance (10.08% women and 18.09% men with outcomes) [10], and the score likely performed better for healthy individuals while failing to identify many at-risk patients early, potentially missing opportunities for interventions that could have improved outcomes [12-14].

For this reason, calibration measures are essential complements to discrimination, since high AUROC values do not necessarily guarantee reliable probability estimates or clinical applicability [6,15,16]. More recent cardiovascular risk prediction models, such as SCORE2, incorporated improved calibration across European populations, but their performance reporting still relies heavily on AUROC [17].

This illustrates a broader issue: even when calibration is addressed, discrimination metrics alone can mask poor identification of minority outcomes, underscoring the need for comprehensive evaluation strategies. Additionally, although the Hosmer-Lemeshow test is a commonly used goodness-of-fit test for logistic regression models, it is less suitable for ML models due to its sensitivity to sample size and arbitrary grouping of predicted probabilities [18].

While these limitations are widely recognized in the ML literature, clinical studies continue to prioritize AUROC in model reporting [19-21]. Most discussions on metric limitations remain either theoretical or based on synthetic datasets [22-25]. As a result, model outputs often lack interpretability and applicability for health professionals, limiting their practical relevance for clinical implementation [26,27]. There are few applied studies using large real-world clinical datasets that demonstrate, in concrete terms, how metric selection affects the identification of high-risk patients and subsequent clinical decisions [4,22,23,27].

Recently, Carriero et al [28] reported the challenges posed by imbalanced datasets in predictive modeling, showing that common strategies to deal with class-imbalance issues, such as oversampling and undersampling, may compromise calibration, leading to overestimated risk predictions and systematic bias [28]. These findings highlight the need for evaluation strategies that move beyond AUROC and artificial rebalancing, offering instead a comprehensive assessment of model performance that prioritizes clinical reliability and patient safety.

This study addresses this gap by applying a structured evaluation of predictive model performance in a real-world clinical setting, using a large, multicenter dataset of hospitalized patients with COVID-19 in Brazil. As a case study to illustrate the impact of class imbalance on model evaluation, we developed 2 ML models to predict kidney replacement therapy (KRT) and in-hospital mortality, outcomes with different prevalence levels, and assessed them using metrics that capture different aspects of model performance. Rather than proposing new predictive models, this study focuses on how commonly used performance metrics influence the interpretation of model usefulness in real-world, imbalanced clinical settings. Beyond AUROC, we focused on class-specific precision, recall, and macro-*F*_1_-scores, which, although well-established in data science, remain underutilized in clinical contexts. Additionally, we critically examine how metric selection influences clinical interpretation in imbalanced scenarios, making our findings relevant not only to data scientists but also to health care professionals. In doing so, this study helps bridge the gap between methodological rigor and clinical applicability in predictive model evaluation.

## Methods

### Study Design

**Figure 1.**
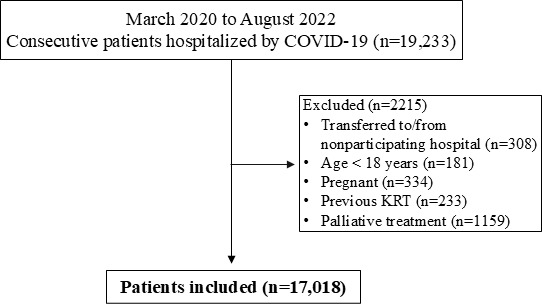
Flowchart of the patients included in the study. KRT: kidney replacement therapy.

This was a retrospective cohort study. We collected data on consecutive adult patients (aged 18 years and older) with laboratory-confirmed COVID-19 [29], admitted in one of 41 participating hospitals in Brazil from March 2020 to August 2022. Details of the cohort have been published elsewhere [30]. Pregnant women; patients undergoing palliative treatment, or with a history of prior KRT or already in KRT upon hospital presentation; and those who were transferred from or to another hospital were excluded from this particular analysis (Figure 1).

Two predictive models were developed and validated: 1 for KRT and 1 for in-hospital mortality. Both models presented imbalanced class distributions, but in different proportions, and were used as case studies. These outcomes were selected due to their clinical relevance and prognostic implications in hospitalized patients with COVID-19 [31].

### Data Collection

Sociodemographic, clinical, and laboratory data; medications; interventions; and outcomes were extracted from medical records by trained researchers using the REDCap (Research Electronic Data Capture) electronic platform [32,33], hosted at the Telehealth Center of the University Hospital of the *Universidade Federal de Minas Gerais* [34,35]. An automated data verification algorithm was implemented to ensure data quality, checking for inconsistencies. Any discrepancies were resolved in consultation with the coordinating researchers.

### Predictors and Outcome Definition

Candidate predictors were selected based on clinical relevance, prior literature, and data availability (MultimediaAppendices 1  2). No automated feature selection was applied, as the study objective was not to maximize predictive performance but to assess how different evaluation metrics behave under identical modeling conditions. The same predictor set was maintained across all experiments to ensure comparability between models and evaluation strategies. KRT was defined as the initiation of dialysis during hospitalization, excluding patients with preexisting chronic dialysis. In-hospital mortality refers to death occurring during hospitalization, as documented in medical records. Patients were classified into binary outcome groups for each end point (KRT vs no KRT; death vs survival).

### The Predictive Models

Extreme gradient boosting (XGBoost) was chosen due to its strong performance in structured clinical data, ability to capture nonlinear relationships, native handling of missing values, and favorable calibration properties reported in prior studies [36-38]. Since XGBoost supports missing values natively, no imputation method was used in the primary analysis.

### Cross-Validation and Modeling Pipeline

A 10-fold stratified cross-validation strategy was used. In each iteration, 1-fold was held out as the test set, while the remaining 9-folds constituted the training set. Within this training partition, a further split was performed to create a validation subset used exclusively for hyperparameter tuning and model selection. All preprocessing and rebalancing procedures that did not require imputation, which are detailed in the next subsection, were performed strictly within the training data of each fold. The test set remained fully held out and was used only for final performance evaluation, preserving the original data distribution and preventing data leakage.

Three key hyperparameters were systematically explored: (1) booster (gbtree, gblinear, and dart), which defines the base learner used to build the ensemble; (2) eta (learning rate), which controls the step-size shrinkage during boosting to prevent overfitting by making the learning process more conservative; and (3) max_depth, which controls the maximum depth of individual trees, thereby regulating model complexity. The complete grid of values evaluated for each hyperparameter is reported in Multimedia Appendix 3.

**Figure 2.**
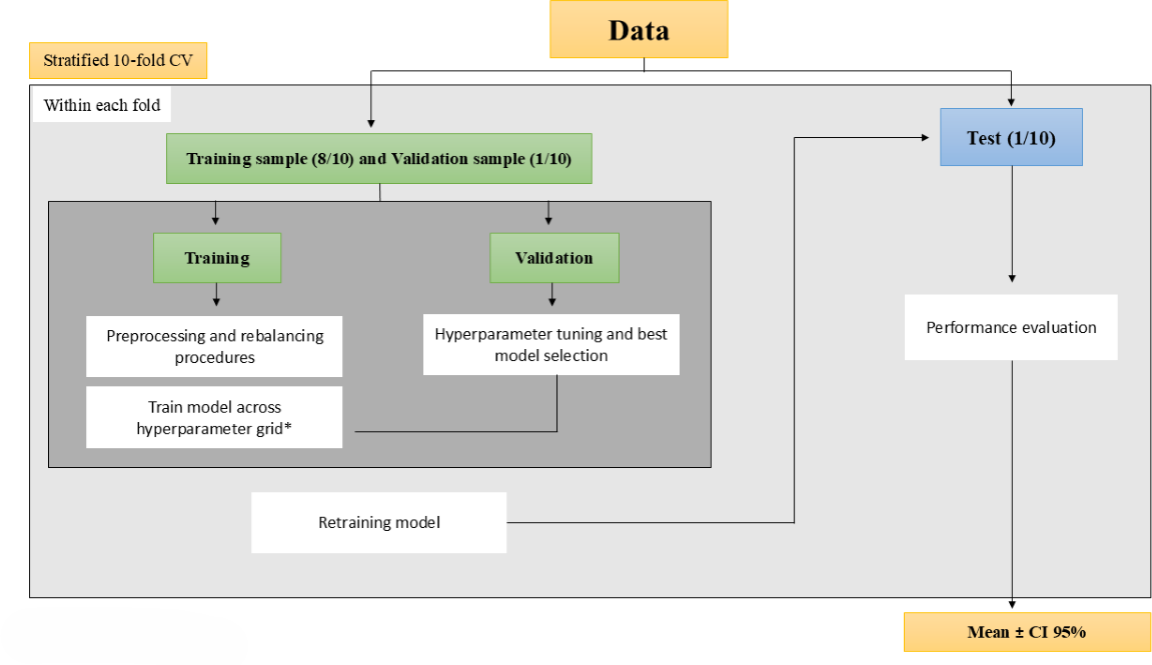
Overview of the analytical pipeline applied within each cross-validation iteration. *booster, eta, max_depth. CV: cross-validation.

After selecting the optimal hyperparameter configuration, the model was retrained on the full training data (training + validation) following standard practice and prior work [39,40], as well as the default behavior of widely used libraries such as scikit-learn (GridSearchCV with refit=True). The held-out test fold was then used exclusively for final performance evaluation. This process was repeated across all folds, so that each fold served once as the test set, and the reported results correspond to the average performance across the 10 iterations. This strategy ensures robust performance estimation and minimizes data leakage [41,42]. The overview of the analytical pipeline is presented in Figure 2.

### Handling of Class Imbalance

To assess the impact of data imbalance on model performance, both oversampling and undersampling techniques were applied exclusively on the training data. For each method, default resampling parameters were used, without additional tuning, following the standard implementation of each algorithm. Due to the intrinsic operational characteristics of certain resampling methods, a fully balanced (1:1) class distribution was not always achieved. The final class proportions after resampling are presented in Multimedia Appendix 3.

Oversampling techniques included Random Oversampling [43], Adaptive Synthetic [44], Synthetic Minority Oversampling Technique (SMOTE) [45], BorderlineSMOTE [46], SVMSMOTE [47], and KMeansSMOTE [48], which increase minority-class representation through random duplication or synthetic sample generation [43,49]. Undersampling methods included Random Undersampling [44], Redundancy-Based Undersampling [39], e2sc-us (Effective, Efficient, and Scalable Confidence-Based-UnderSampling) [39], Condensed Nearest Neighbor [50], Near Miss 1 [51], and Near Miss 2 [51], which reduce majority-class instances while attempting to preserve relevant decision boundaries [39].

Because most resampling algorithms cannot handle missing values, the MissForest imputation method was incorporated into the pipeline when required, exclusively within the training data for rebalancing experiments [52]. Importantly, the imputation model was fitted exclusively on the training partition within each cross-validation fold and subsequently applied to the corresponding validation and test sets, thereby preventing information leakage.

For each resampling strategy, the entire modeling pipeline, including imputation (when applicable), resampling, and model training, was re-executed within each cross-validation iteration. Hyperparameter tuning was performed from scratch for each resampled dataset, ensuring that model optimization was specific to each data configuration. Details of the hyperparameter search space are provided in Multimedia Appendix 4. No information from the test set was used at any stage of model development, including imputation, resampling, or hyperparameter tuning.

To enable direct comparison across models, a fixed probability threshold of 0.5 was used for classification. This threshold was selected based on its consistent performance across preliminary analyses. All experiments were conducted using a fixed random seed (random_state=42), applied consistently across all stochastic components of the pipeline, including cross-validation splitting, imputation, resampling procedures, and model initialization, ensuring full reproducibility.

### Performance Evaluation

Model performance was assessed using a complementary set of metrics that offer different perspectives to evaluate model performance, capturing global discrimination, class-specific behavior, calibration, and clinical usefulness (MultimediaAppendices 5  6). All metrics were computed on the held-out test fold in each iteration and averaged across folds. Specifically, we analyzed global metrics, such as accuracy and AUROC, as well as metrics that are more sensitive to class imbalance, such as macro-*F*_1_ and per-class precision and recall, including precision and recall across both majority and minority classes and the impact of different decision thresholds on the values of these metrics.

We first evaluated global performance using accuracy and AUROC. These metrics summarize overall discrimination across all instances but treat all elements equally, regardless of their class, which inherently biases these metrics toward the majority class in imbalanced datasets [2,4]. Accuracy represents the proportion of correctly classified instances [4], while AUROC quantifies the model’s ability to rank positive cases higher than negative ones across all decision thresholds [6,11]. However, what is considered a “correct” prediction depends on the chosen decision threshold for the risk score, as different thresholds influence the balance between sensitivity and specificity [4].

To explicitly capture performance under class imbalance, we additionally reported per-class precision, recall, and *F*_1_-score, as well as macro-*F*_1_, which assigns equal weight to each class regardless of prevalence [50,51,53]. To better characterize performance under class imbalance, we first examined class-specific precision and recall, which directly quantify errors for both minority and majority outcomes. Regarding the positive class, recall (sensitivity) reflects the proportion of true cases correctly identified, whereas precision (positive predictive value) reflects the proportion of predicted positives that are true events. The same logic applies to the negative class, where recall corresponds to specificity and precision to negative predictive value [54,55].

In addition to these class-specific metrics, we evaluated resampling strategies using TPRGap, a bias-oriented measure that quantifies performance disparity between classes as the absolute difference between their true-positive rates. This metric directly captures classifier favoritism toward the majority class, which may persist even when global performance measures remain high [56]. Finally, to summarize the trade-off between precision and recall in a single indicator, we reported the *F*_1_-score and its macroaveraged form, which assigns equal weight to each class and is therefore robust to outcome imbalance [50,51,54,57].

While this perspective is common in the ML literature, it may be less intuitive for health care professionals, who are generally more familiar with metrics such as sensitivity and specificity. By reporting both precision and recall for each class, we provide a more nuanced and clinically interpretable understanding of model performance, especially relevant in the presence of class imbalance [50,51,54,57]. This approach enables assessment not only of how well the model identifies patients at risk but also how confidently it excludes those unlikely to experience the outcome. Therefore, it supports a more comprehensive assessment of predictive usefulness and more informed decision-making in clinical applications.

Primary analyses were conducted using a default probability threshold of 0.5, consistent with standard binary classification practice. To explore clinically relevant trade-offs between missed events and false alarms, we further evaluated precision-recall behavior across varying decision thresholds using precision-recall curves [50,56]. The precision-recall curve was generated by plotting precision against recall at various decision thresholds [54].

Model calibration was assessed using the plot with predicted probability against observed probability, testing intercept equals zero and slope equals 1. In a well-calibrated model, there is agreement between observed and predicted events, allowing the probability to be interpreted as the confidence in the prediction [58,59]. In addition, the global accuracy of the model was assessed using the Brier score. The Brier score ranges from 0 to 1, with lower values indicating better probabilistic accuracy [15].

Clinical usefulness was assessed through decision curve analysis, which quantifies net benefit across a range of decision thresholds compared with “treat-all” and “treat-none” strategies [55,57]. While decision curves assess whether model-guided decisions outperform simple strategies, they do not ensure balanced error distribution or detect bias toward the majority class, reinforcing the need for class-specific performance metrics [55,57]. Finally, the learning curves were used as a graphical representation of how a model’s performance evolves as training data are added [60].

### Risk-of-Bias Assessment and Reporting

This study adheres to the TRIPOD+AI (Transparent Reporting of a multivariable prediction model for Individual Prognosis Or Diagnosis + Artificial Intelligence) standards for transparent reporting (Multimedia Appendix 7) [6]. To ensure methodological rigor, we used the PROBAST+AI (Updated Quality, Risk of Bias, and Applicability Assessment Tool for Prediction Models Using Regression or Artificial Intelligence Methods) to assess risk of bias and applicability (Multimedia Appendix 8). The study was considered to have a low risk of bias in all domains (participants, predictors, outcomes, and analysis). However, the lack of external validation of the model should be considered as a point of attention in the domain of analysis. Applicability concerns were judged to be low across all domains [16].

### Ethical Considerations

The study was approved by the Brazilian National Research Ethics Committee—Comissão Nacional de Ética em Pesquisa (CAAE 30350820.5.0000.0008) and internal approval of ethics boards from each hospital. Individual informed consent term was waived due to the pandemic situation and analysis of deidentified data, based on chart review only.

## Results

### Overview

**Figure 3.**
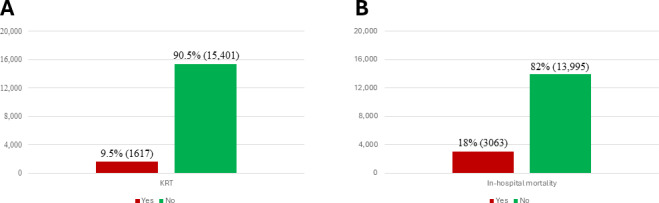
Imbalanced outcome class distribution. (A) KRT; (B) in-hospital mortality. KRT: kidney replacement therapy.

The database included 17,018 patients (median age of 60 years, IQR 37‐83; 54.6% were men). The outcome distributions were highly imbalanced (Figure 3). Approximately 9.5% (1617/17,018) of the patients underwent KRT (1617 patients), resulting in an imbalance ratio of 9.5:1. Similarly, 18% (3063/17,018) of the patients died, corresponding to an imbalance ratio of 4.6:1.

### Prediction of KRT

The predictive XGBoost model demonstrated high overall performance when considering accuracy (0.910) and AUROC (0.928; Table 1). However, due to class imbalance, the other metrics revealed some important observations that would otherwise be overlooked. Notably, the macro-*F*_1_-score of 0.695 suggests relatively lower performance, specifically for the minority class (KRT=yes; Tables1  2). This is mainly due to the low recall (0.372), indicating that the model struggles to correctly identify a large proportion of actual KRT cases. The precision for this class was 0.539, resulting in an *F*_1_-score of 0.439 (Table 2).

**Table 1.** Global metrics at different cutoff thresholds for kidney replacement therapy^a^.

Cutoff	Accuracy	AUROC^b^	Macro-*F*_1_	Brier
50%	0.910 (0.906‐0.914)	0.928 (0.923‐0.933)	0.695 (0.682‐0.708)	0.066 (0.063‐0.069)
40%	0.907 (0.902‐0.912)	0.930 (0.923‐0.937)	0.711 (0.694‐0.728)	0.062 (0.060‐0.064)
30%	0.901 (0.896‐0.906)	0.930 (0.923‐0.937)	0.731 (0.719‐0.743)	0.062 (0.060‐0.064)
20%	0.890 (0.884‐0.896)	0.930 (0.923‐0.937)	0.742 (0.729‐0.755)	0.062 (0.060‐0.064)
10%	0.865 (0.861‐0.869)	0.930 (0.923‐0.937)	0.731 (0.723‐0.739)	0.062 (0.060‐0.064)

a Data are presented as mean (95% CI).

b AUROC: area under the receiver operating characteristic curve.

**Table 2.** Per-class metrics at different cutoff thresholds for kidney replacement therapy^a^.

Cutoff	Precision		Recall		*F*_1_	
KRT^b^	No KRT	KRT	No KRT	KRT	No KRT
50%	0.539 (0.508‐0.570)	0.936 (0.934‐0.938)	0.372 (0.345‐0.399)	0.966 (0.961‐0.971)	0.439 (0.415‐0.463)	0.951 (0.949‐0.953)
40%	0.511 (0.481‐0.541)	0.942 (0.938‐0.946)	0.442 (0.406‐0.478)	0.956 (0.953‐0.959)	0.474 (0.442‐0.506)	0.949 (0.946‐0.952)
30%	0.480 (0.460‐0.500)	0.953 (0.950‐0.956)	0.563 (0.536‐0.590)	0.936 (0.933‐0.939)	0.518 (0.495‐0.541)	0.945 (0.942‐0.948)
20%	0.449 (0.430‐0.468)	0.967 (0.963‐0.971)	0.701 (0.668‐0.734)	0.910 (0.905‐0.915)	0.547 (0.525‐0.569)	0.937 (0.934‐0.940)
10%	0.400 (0.390‐0.410)	0.981 (0.978‐0.984)	0.837 (0.817‐0.857)	0.868 (0.864‐0.872)	0.542 (0.529‐0.555)	0.921 (0.918‐0.924)

a Data are presented as mean (95% CI).

b KRT: kidney replacement therapy.

**Figure 4.**
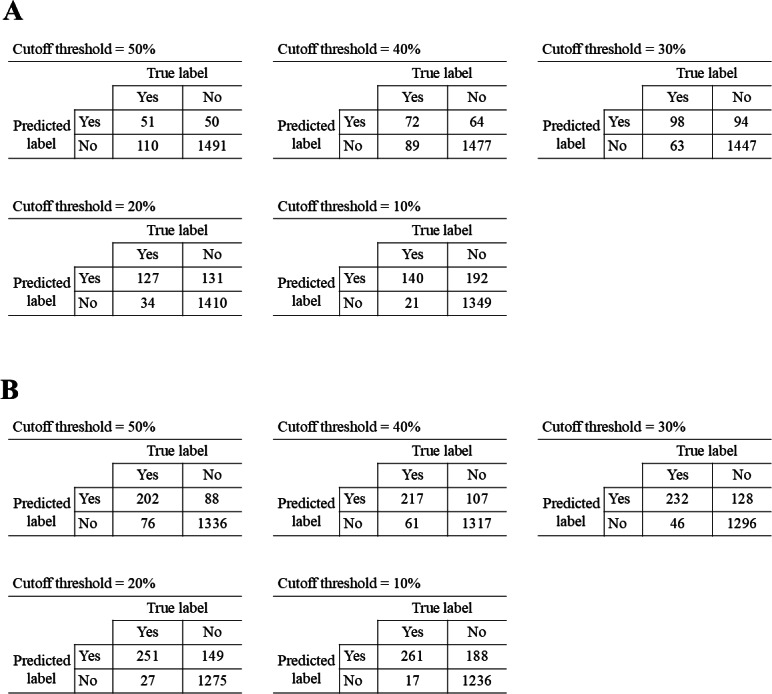
(A) Confusion matrix at different cutoff thresholds to predict kidney replacement therapy. (B) Confusion matrix at different cutoff thresholds to predict in-hospital mortality.

The confusion matrices for KRT prediction also highlight this trend, showing that lowering the threshold from 50% to 10% enhances sensitivity, evidenced by a 174.5% increase in true positive (from 51 to 140). However, this adjustment also leads to a 284.0% rise in false positive (from 50 to 192; Figure 4A).

Changing the cutoff threshold affected the model’s precision, recall, and *F*_1_ values. In this context, considering the class of interest, which is KRT, and the default cutoff threshold of 50%, the precision was 0.539, recall was 0.372, and *F*_1_-score was 0.439 (Table 2). Lowering the cutoff threshold to 20% resulted in a precision of 0.449, recall of 0.701, and an improved *F*_1_-score of 0.547 (Table 2). The data presented in Multimedia Appendix 9 elucidate the trade-off between precision and recall, where an increase in precision usually implies a reduction in recall and vice versa.

**Figure 5.**
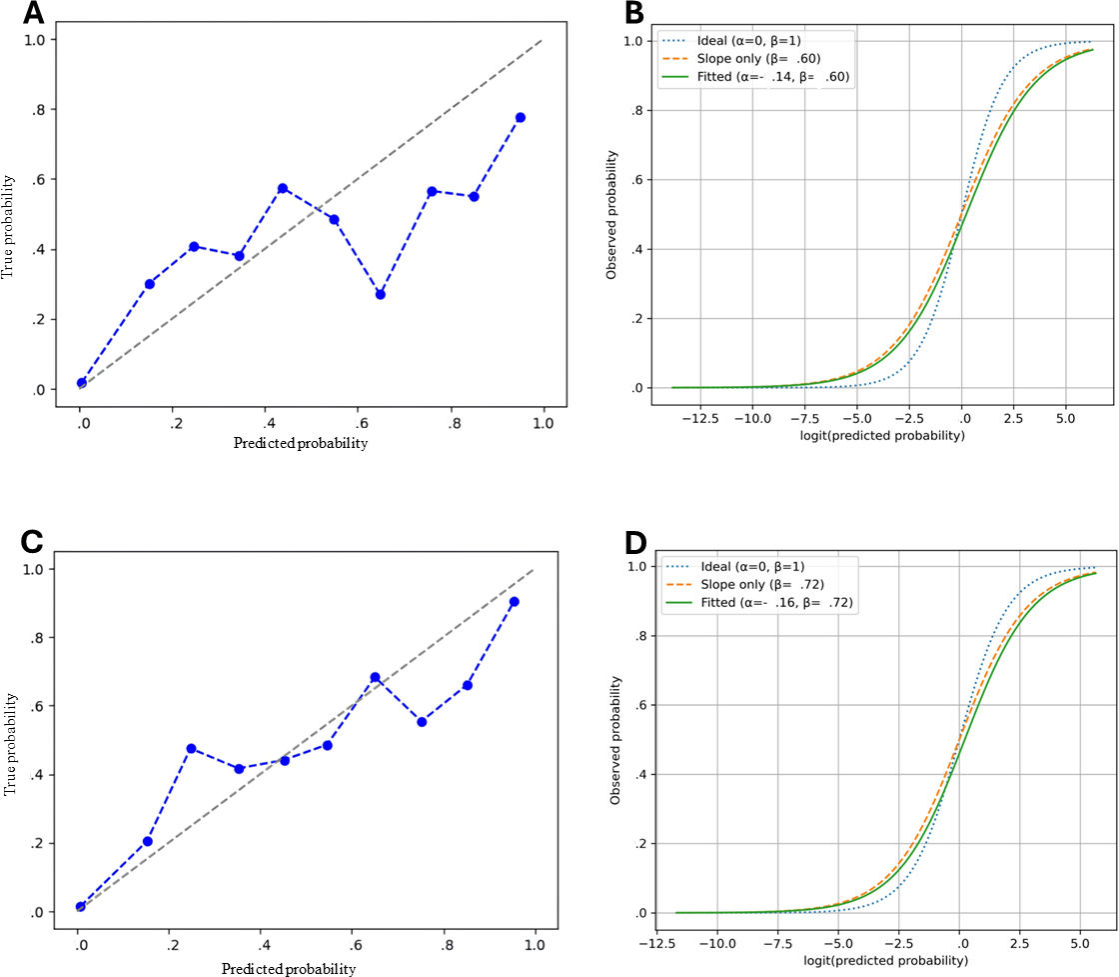
(A). Calibration curve for kidney replacement therapy. (B). Plot showing the calibration slope and intercept for the kidney replacement therapy task. (C). Calibration curve for death. (D). Plot showing the calibration slope and intercept for the death.

The calibration plot shows systematic deviation from the diagonal, with predicted probabilities falling below the diagonal at higher values and above it at lower values, indicating overconfidence at the extremes (Figure 5A). This pattern is further supported by a calibration slope of 0.60 and an intercept of −0.14 (Figure 5B), indicating both overconfident predictions and a slight global overestimation of risk.

**Figure 6.**
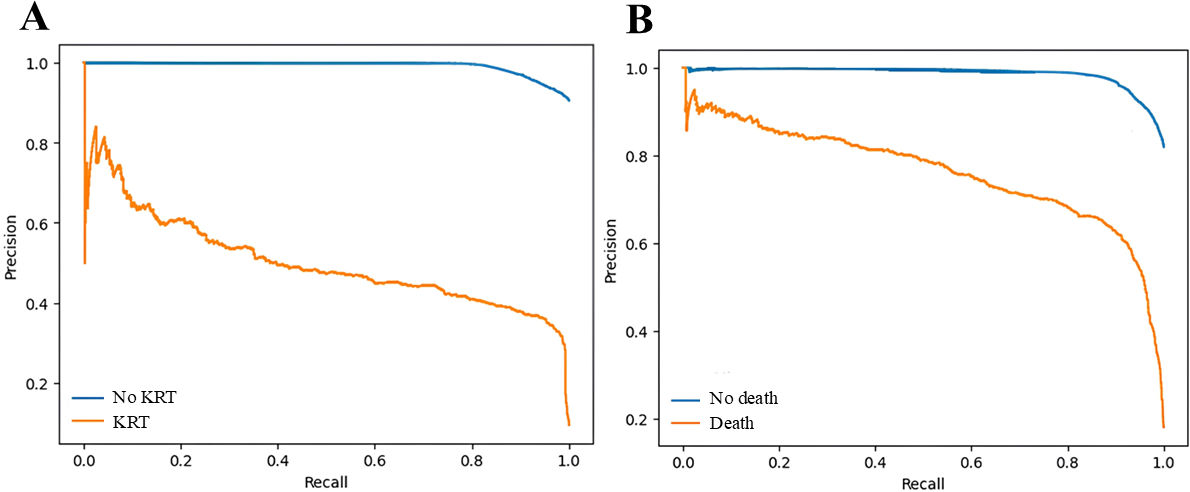
(A) Precision-recall curves for patients undergoing KRT and not undergoing KRT. (B) Precision-recall curves for death, and no death. KRT: kidney replacement therapy.

The precision-recall curve for each class (Figure 6A) showed that the non-KRT class achieved high precision and recall simultaneously, which is desirable, while the KRT class showed a performance closer to random. In other words, the model is relatively good at recalling non–KRT patients (high specificity), but it struggles to identify the ones who underwent KRT (low sensitivity).

It is observed that the curve for the class that did not undergo KRT remains close to the upper right corner, indicating that the model can achieve high precision and recall rates simultaneously. Conversely, the curve for the (interest) minority class (underwent KRT) approaches the diagonal, suggesting that the model is struggling to balance precision and recall, with performance close to random.

The decision curve analysis (Multimedia Appendix 10) indicates that the proposed model generates a positive net benefit for low to moderate decision thresholds, starting at approximately 0.10 and gradually decreasing to zero as the threshold increases to 0.5. Consequently, the model exhibits a practical benefit for decision thresholds (*P≤*.5), indicating usefulness in scenarios that tolerate decisions based on relatively moderate predicted probabilities. In contrast, the strategy of treating all cases shows a positive net benefit only at very low thresholds, reaching zero around a threshold of 0.10 and becoming increasingly negative thereafter.

### Prediction of In-Hospital Mortality

The predictive XGBoost model achieved high values of accuracy (0.900) and AUROC (0.945; Table 3). However, due to class imbalance, the macro-*F*_1_-score of 0.830 indicates lower performance (Table 3). Specifically, for the minority class (deceased=yes), the precision is 0.725, the recall is 0.718, and the *F*_1_-score is 0.721 (Table 4).

**Table 3.** Global metrics at different cutoff thresholds for death^a^.

Cutoff	Accuracy	AUROC^b^	Macro-*F*_1_	Brier
50%	0.900 (0.896‐0.904)	0.945 (0.939‐0.951)	0.830 (0.825‐0.835)	0.072 (0.069‐0.075)
40%	0.900 (0.895‐0.905)	0.945 (0.939‐0.951)	0.837 (0.830‐0.844)	0.072 (0.069‐0.075)
30%	0.896 (0.891‐0.901)	0.945 (0.939‐0.951)	0.837 (0.829‐0.845)	0.072 (0.069‐0.075)
20%	0.893 (0.887‐0.899)	0.945 (0.939‐0.951)	0.840 (0.831‐0.849)	0.072 (0.069‐0.075)
10%	0.878 (0.870‐0.886)	0.945 (0.939‐0.951)	0.827 (0.815‐0.839)	0.072 (0.069‐0.075)

a Data are presented as mean (95% CI).

b AUROC: area under the receiver operating characteristic curve.

The confusion matrices for mortality prediction demonstrated a similar precision-recall trade-off (Figure 4B). Reducing the cutoff threshold from 50% to 10% enhanced recall, with true positives increasing by approximately 29.1% (from 202 to 261), but also resulted in a 113% increase in false positives (from 88 to 188).

As with KRT, the cutoff threshold affected the precision, recall, and *F*_1_-scores. At a 50% cutoff threshold, the precision is 0.725, the recall is 0.718, and the *F*_1_-score is 0.721 (Table 4). Lowering the threshold to 20% increased the recall (0.880) and improved the *F*_1_-score (0.748), while precision decreased to 0.651 (Table 4 and Multimedia Appendix 4). Similar to KRT, the calibration plot (slope=0.72; intercept=−0.16) was not satisfactory, and the Brier score was low (0.072; Figure 5C and D and Table 3).

The precision-recall curves for each class (Figure 6B) followed a similar pattern to that observed for KRT, with the non–death class exhibiting higher precision and recall than the death class. Once again, the model performed well in identifying survivors (high specificity) but demonstrated limited ability to detect patients who died (low sensitivity).

It is observed that the curve for the non–death class remains close to the upper right corner, indicating that the model can achieve high precision and recall rates simultaneously. In contrast, the curve for the death class, which is the minority class and of greater interest, approaches the diagonal (random model).

Similar to KRT, the decision curve for death shows that the net benefit of the strategy of treating all cases is approximately 0.2, while the proposed model achieves a substantially higher net benefit, around 0.8 (Multimedia Appendix 11). This behavior demonstrates that indiscriminate intervention quickly becomes inadequate as the decision threshold increases, while the proposed model maintains its practical usefulness over a substantially wider range of thresholds.

**Table 4.** Per-class metrics at different cutoff thresholds for death^a^.

Cutoff	Precision		Recall		*F*_1_	
Death	No death	Death	No death	Death	No death
50%	0.725 (0.703‐0.747)	0.938 (0.934‐0.942)	0.718 (0.710‐0.726)	0.940 (0.934‐0.946)	0.721 (0.712‐0.730)	0.939 (0.937‐0.941)
40%	0.701 (0.678‐0.724)	0.949 (0.946‐0.952)	0.776 (0.768‐0.784)	0.927 (0.920‐0.934)	0.736 (0.725‐0.747)	0.938 (0.935‐0.941)
30%	0.673 (0.652‐0.694)	0.958 (0.955‐0.961)	0.821 (0.811‐0.831)	0.912 (0.906‐0.918)	0.739 (0.726‐0.752)	0.935 (0.931‐0.939)
20%	0.651 (0.630‐0.672)	0.971 (0.969‐0.973)	0.880 (0.872‐0.888)	0.896 (0.889‐0.903)	0.748 (0.734‐0.762)	0.932 (0.928‐0.936)
10%	0.608 (0.585‐0.631)	0.980 (0.977‐0.983)	0.921 (0.910‐0.932)	0.869 (0.861‐0.877)	0.732 (0.714‐0.750)	0.921 (0.916‐0.926)

a Data are presented as mean (95% CI).

### The Influence of Class Imbalance on Prediction

The model for KRT, which exhibited higher class imbalance, demonstrated superior performance for accuracy and AUROC when compared with the model for mortality. However, when examining the precision and recall for the minority class, the performance was suboptimal, and the KRT model exhibited lower performance than the mortality model. This pattern was also reflected in the macro-*F*_1_-score, where the KRT model displayed a more significant drop in performance, further highlighting the impact of class imbalance.

It is important to highlight that KRT represents a distinct endpoint from mortality. Although both models used the same set of variables, the features and the importance of each feature vary depending on the endpoint (MultimediaAppendices 12  13). Therefore, discrepancies in the performance of the KRT and mortality models should not be solely attributed to differences in class balance.

### Learning Curves Analysis

**Figure 7.**
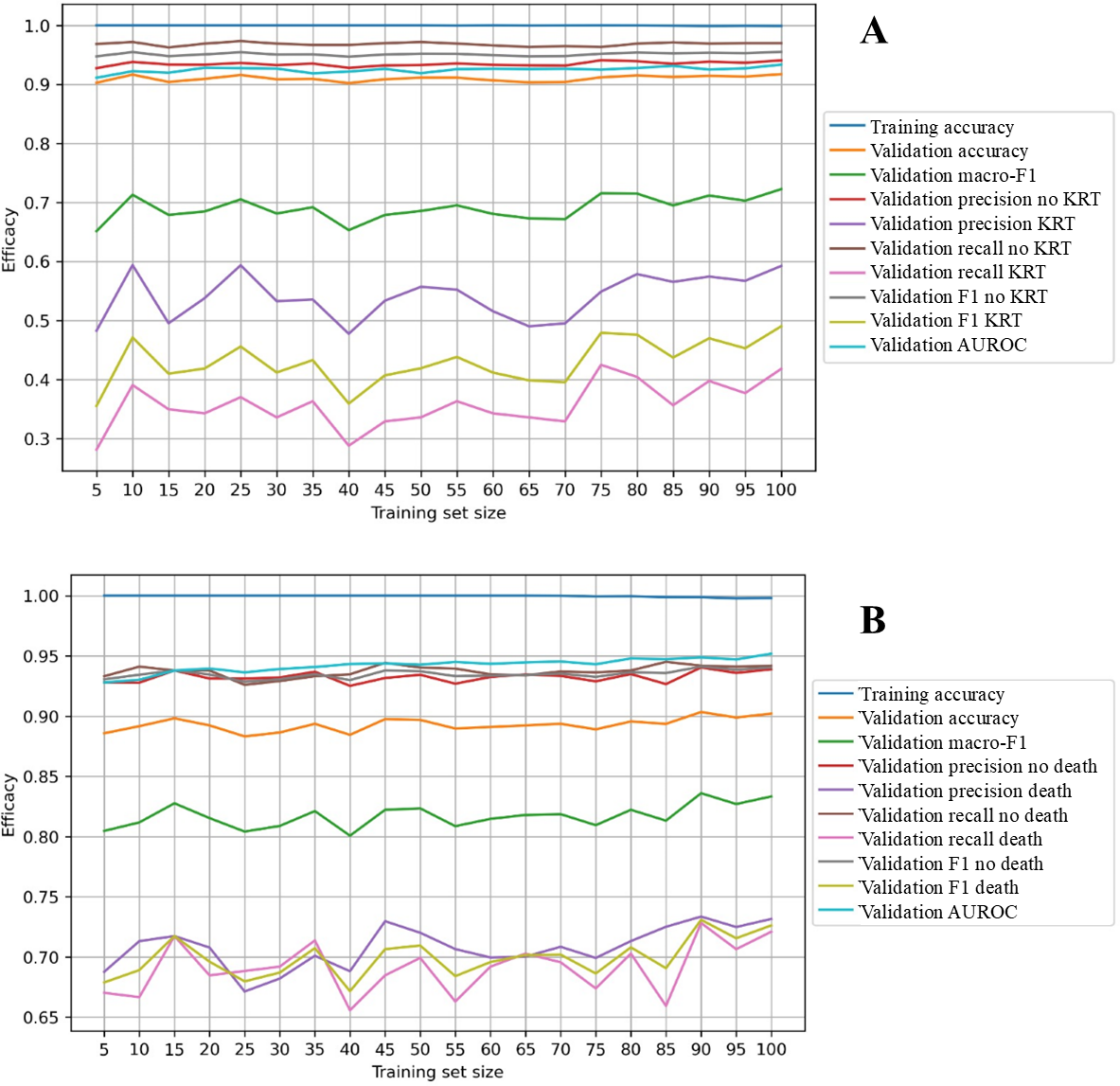
(A) Learning curves for different training set sizes for kidney replacement therapy. (B) Learning curves for different training set sizes for death. AUROC: area under the receiver operating characteristic curve; *F*_1_: *F*_1_-score; KRT: kidney replacement therapy.

The learning curves show a plateau-like shape, with stable validation performance across all training set sizes for both outcomes (Figures 7A and 7B). This pattern suggests limited change in performance as the training set size increases, indicating that model performance does not substantially improve with additional data.

### Impact of Balancing Strategies on Prediction Results and on the Metrics

When evaluating the impact of class rebalancing strategies, we observe a clear and systematic discrepancy between AUROC and metrics that explicitly account for class-specific behavior. As shown in MultimediaAppendices 12  13, AUROC remains consistently high across all experimental conditions, exceeding 0.8 in every scenario, regardless of whether rebalancing is applied or whether minority-class performance substantially improves or deteriorates. This stability may give the misleading impression that rebalancing strategies have limited effect on model behavior.

However, a closer inspection using alternative metrics reveals a markedly different picture. In particular, TPRGap provides a direct measure of classifier bias induced by class imbalance, capturing disparities in true-positive rates between classes. Using this metric, several rebalancing techniques substantially reduce bias relative to the unbalanced baseline. For instance, in the death outcome, TPRGap decreases from 0.230 in the unbalanced setting to 0.043 when using Redundancy-Based Undersampling, indicating a pronounced reduction in class-dependent performance disparity. In contrast, AUROC changes only marginally in the same scenario, from 0.945 to 0.941, failing to reflect this improvement.

Similar patterns are observed across other class-aware metrics, particularly positive-class precision, recall, and *F*_1_-score. These metrics exhibit significant sensitivity to rebalancing strategies, capturing both beneficial and harmful effects on minority-class performance. In several cases, rebalancing leads to meaningful gains in recall at the expense of precision, or vice versa, reflecting trade-offs that are critical in clinical decision-making contexts. Yet, AUROC remains largely unchanged, masking these trade-offs and providing little insight into how the classifier’s behavior actually shifts.

An especially illustrative example arises in the death outcome under the Near Miss 2 undersampling strategy. In this case, the positive-class *F*_1_-score drops sharply from 0.721 in the unbalanced model to 0.345, signaling a severe degradation in clinically relevant performance. Despite this substantial decline, AUROC remains comparatively high, decreasing from 0.945 to 0.809. This modest reduction does not adequately reflect the magnitude of the performance loss experienced by the minority class, underscoring the disconnect between AUROC and clinically meaningful outcomes.

## Discussion

### Principal Findings

In recent years, the rapid expansion of ML applications in health care has led to an increasing number of predictive models being proposed for clinical use. However, many of these studies continue to rely primarily, or even exclusively, on AUROC to report model performance, even in highly imbalanced clinical scenarios. Our findings demonstrate the limitations of this practice, showing that high AUROC values may coexist with poor performance for clinically critical minority outcomes.

Although methodological literature has long acknowledged the limitations of AUROC and accuracy in imbalanced settings, clinical prediction studies frequently continue to emphasize these global metrics. By applying complementary class-specific measures and analyzing the learning curves in a large cohort of over 17,000 hospitalized patients with COVID-19, our study provides practical evidence of how metric selection directly influences clinical interpretation, particularly when outcomes are imbalanced.

For both KRT and in-hospital mortality, AUROC values suggested excellent discrimination. However, class-specific metrics revealed substantial deficiencies in identifying minority outcomes. The KRT model achieved an AUROC of 0.928. However, recall for the KRT class was only 0.372, meaning that the model failed to identify nearly two-thirds of patients who would require dialysis. In a real clinical scenario, such as a hospital without dialysis services, this could result in missed opportunities for early referrals, with serious consequences for patient care. This discrepancy was captured by the macro-*F*_1_-score (0.695), which penalizes imbalanced performance across classes and thus offers a more clinically realistic summary of the model’s performance than AUROC alone.

Calibration analysis further highlighted these limitations. Calibration slopes of 0.60 and 0.72, along with negative intercepts, indicate suboptimal calibration for both outcomes. Although Brier scores were low, this likely reflects strong discrimination combined with outcome imbalance, rather than well-calibrated probability estimates. These findings reinforce that no single metric adequately captures model performance in imbalanced clinical settings.

Precision-recall analysis further highlighted the limitations of AUROC-based evaluation by prioritizing performance on the minority class, which often represents the clinically most relevant outcome [57]. While decision curve analysis further illustrates that clinically meaningful usefulness may vary substantially across thresholds [61,62] and demonstrated net benefit across certain thresholds, it did not capture how prediction errors were distributed between classes. By examining class-specific precision and recall across varying thresholds, we directly linked model behavior to real-world clinical trade-offs between underdiagnosis and overdiagnosis. Given that class prevalence directly affects metrics such as precision and recall, particularly in imbalanced settings [11,61,63], relying on AUROC alone may obscure clinically relevant deficiencies in minority-class performance. These results underscore the importance of reporting complementary, class-aware metrics, as no single metric adequately captures model performance across different clinical contexts. Therefore, metric selection should be guided by the intended clinical application. In high-risk settings, maximizing recall may be preferable to avoid missing cases, even at the expense of increased false positives. In contrast, in resource-limited settings, higher precision may be prioritized to reduce unnecessary interventions. These trade-offs cannot be adequately captured by a single metric, reinforcing the need for a multidimensional evaluation approach.

Learning curve analysis provided an additional and complementary perspective on model performance. In general, learning curves may reflect either progressive improvement with increasing data or early convergence in relatively simple prediction tasks [60]. In our study, however, the combination of flat learning curves, persistently low minority-class performance, and stable AUROC values across increasing training set sizes suggests limited gains in performance as more data are added, highlighting that AUROC alone may not capture important aspects of model behavior in imbalanced settings.

This finding has relevant methodological considerations. Despite consistently high AUROC values (>0.92), the lack of substantial improvement in performance with increasing training data suggests that discrimination alone may not fully reflect how model performance evolves, particularly for identifying high-risk patients. In this context, AUROC may reflect stable ranking ability driven by dataset characteristics, such as class imbalance, rather than improvements in clinically relevant performance. Therefore, reliance on AUROC alone may lead to overestimation of model performance and potential underrecognition of high-risk patients.

Learning curves offer a complementary tool to assess how model performance changes as additional data are incorporated [60]. When performance remains relatively stable, caution is warranted in interpreting high discrimination metrics as sufficient evidence of model adequacy. Together, these findings reinforce that AUROC alone is insufficient to determine whether a model is suitable for clinical use, particularly in imbalanced scenarios.

International reporting frameworks such as PROBAST+AI and TRIPOD+AI emphasize comprehensive evaluation of predictive model performance and transparency in results presentation but remain focused on global performance measures of discrimination, calibration, and overall clinical usefulness [6,16]. Although these frameworks acknowledge the impacts of class imbalance on outcomes, they do not offer strategies for measuring the redistribution of errors across classes with varying thresholds, nor do they recommend incorporating learning curve analysis into model evaluation [6,16].

Additionally, according to PROBAST+AI, applicability concerns were considered low, as the study population, predictors, and outcomes are consistent with real-world clinical settings. However, this should not be interpreted as evidence supporting clinical use of the models. Despite this apparent applicability, the models demonstrated important limitations in clinically relevant performance, including suboptimal calibration and limited sensitivity for the minority class. This apparent contradiction highlights a key finding of our study: even when models are developed using appropriate data and aligned with clinical contexts, reliance on conventional metrics such as AUROC may obscure critical weaknesses. Therefore, methodological soundness and contextual relevance alone are insufficient to ensure clinically meaningful performance, reinforcing the need for comprehensive, class-aware evaluation frameworks before considering any potential clinical implementation.

Our findings provide a methodological contribution that extends beyond these current standards. Specifically, we demonstrate that high AUROC values were maintained despite limited changes in performance across increasing training set sizes. This observation suggests that AUROC alone may not reflect whether a model has learned clinically meaningful patterns but rather may capture stable discrimination driven by dataset characteristics such as class imbalance, highlighting the need for more transparent and comprehensive reporting.

This has important implications: models may comply with current reporting standards while still underperforming in clinically relevant minority outcomes, which are often the most clinically relevant. Because learning curves are rarely reported, this limitation may go unrecognized in many published models. Incorporating learning curve analysis alongside class-specific metrics can therefore enhance transparency and provide a more robust assessment of model performance.

Resampling techniques, including over- and undersampling, resulted in only modest improvements in minority-class performance and did not resolve the fundamental limitations of AUROC-based evaluation [28,62,64,65]. Even when class distributions were artificially modified, AUROC remained largely insensitive to clinically meaningful changes in recall and precision. This reinforces that rebalancing alone cannot compensate for inappropriate performance metrics.

Our study contributes to literature by bridging theoretical concerns with practical, real-world application. By evaluating 2 predictive models in a large clinical cohort with varying degrees of outcome imbalance, we demonstrate how metric selection and threshold choice directly influence clinical interpretation and link how these shifts directly affect clinical decision-making. By evaluating per-class precision and recall across multiple cutoffs and visualizing these relationships through precision-recall curves, we make explicit the trade-offs inherent to real-world model deployment.

These trade-offs are particularly relevant in health care, where underdiagnosing high-risk patients (low recall) may lead to missed interventions, while overdiagnosis (low precision) can result in unnecessary procedures and resource strain [66]. In high-stakes settings, such as intensive care units or emergency triage, prioritizing recall may be appropriate, even at the cost of more false positives, to avoid missing patients at risk of deterioration. On the other hand, in resource-constrained environments, higher precision may be preferable. Together, these findings reinforce that no single metric is sufficient and that different clinical contexts require different operating points and different emphases on recall, precision, or their balance, which is effectively summarized by the macro-*F*_1_.

Despite growing methodological awareness [4,6-9,50,64], most applied health care research still relies predominantly on this AUROC for model evaluation (MultimediaAppendices 14  15) [67]. For example, DynaMed, a widely used evidence-based clinical reference platform, currently lists 26 predictive models specifically developed for COVID-19—1 diagnostic and 25 prognostics, including outcomes such as severe disease progression, thrombosis, intensive care unit admission, KRT, and mortality (Multimedia Appendix 11) [67]. Notably, 88.5% (23/26) of these models primarily report AUROC as the main performance metric [67].

Additionally, some studies report multiple metrics without adequately contextualizing their relevance or the trade-offs involved [67-69]. Our findings highlight the importance of not only reporting multiple metrics but also interpreting them in relation to clinical context and outcome imbalance.

Therefore, metric selection and learning curve analysis may substantially influence the clinical interpretation of model performance. Choosing evaluation strategies that account for outcome imbalance and clinical priorities is essential to support more rigorous evaluation before potential clinical implementation of predictive models.

### Limitations

The methodology focused on a single algorithm (XGBoost), although the observed patterns are not model-specific and reflect broader issues related to class imbalance and performance evaluation. External validation was not feasible due to data availability; however, this does not affect the central methodological contribution of the study, which concerns the interpretation of model performance rather than the generalizability of a specific model. Therefore, our findings should not be interpreted as supporting the clinical use of the models presented, given the lack of external validation and suboptimal calibration. Importantly, the aim of this study was not to develop the best-performing predictive model but to examine how evaluation strategies influence the interpretation of model performance in imbalanced clinical scenarios.

In prediction tasks with imbalanced outcomes, which are common in health care, reliance on accuracy and AUROC alone may obscure clinically important failures. Complementary metrics, including precision, recall, and macro-*F*_1_, provide a more realistic assessment of model performance and should be systematically reported. In addition, learning curve analysis offers insight into a model’s learning dynamics and helps explore how model performance evolves as more training data are incorporated. Together, these approaches support a more comprehensive and clinically meaningful evaluation of predictive models, particularly in imbalanced settings, rather than their direct translation into clinical practice.

## Supplementary material

10.2196/86379 Multimedia Appendix 1 Potential predictors for patients with COVID-19 undergoing kidney replacement therapy.

10.2196/86379 Multimedia Appendix 2 Potential predictors for in-hospital mortality in patients with COVID-19.

10.2196/86379 Multimedia Appendix 3 Final proportions in the training partition after application of each rebalancing strategy.

10.2196/86379 Multimedia Appendix 4 Hyperparameters evaluated for optimization using extreme gradient boosting (XGBoost).

10.2196/86379 Multimedia Appendix 5 Definitions and characteristics of the metrics frequently used to evaluate performance in predictive models, using the machine learning and the statistics terminology.

10.2196/86379 Multimedia Appendix 6 Hypothetical confusion matrix and calculation of performance metrics for binary classification.

10.2196/86379 Multimedia Appendix 7 Checklist for TRIPOD+AI (Transparent Reporting of a Multivariable Prediction Model for Individual Prognosis or Diagnosis + Artificial Intelligence).

10.2196/86379 Multimedia Appendix 8 Checklist for PROBAST+AI (Updated Quality, Risk of Bias, and Applicability Assessment Tool for Prediction Models Using Regression or Artificial Intelligence Methods).

10.2196/86379 Multimedia Appendix 9 Means of performance metrics at different cutoffs for kidney replacement therapy (KRT) and not undergoing KRT.

10.2196/86379 Multimedia Appendix 10 Decision curve for kidney replacement therapy.

10.2196/86379 Multimedia Appendix 11 Decision curve for death.

10.2196/86379 Multimedia Appendix 12 Global and per-class metrics for different rebalancing techniques for kidney replacement therapy.

10.2196/86379 Multimedia Appendix 13 Global and per-class metrics for different rebalancing techniques for death.

10.2196/86379 Multimedia Appendix 14 Outcomes and evaluation metrics of predictive scores for patients with COVID-19 based on the DynaMed summary.

10.2196/86379 Multimedia Appendix 15 Outcomes and evaluation metrics of predictive scores for cardiovascular disease based on the DynaMed summary.

10.2196/86379 Multimedia Appendix 16 Means of performance metrics at different cutoffs for death and no death.

10.2196/86379 Multimedia Appendix 17 Features’ importance and contribution to the final predictive model of kidney replacement therapy.

10.2196/86379 Multimedia Appendix 18 Features’ importance and contribution to the final predictive model of in-hospital mortality.

## Abbreviations

AUROC: area under the receiver operating characteristic curve
e2sc-us: Effective, Efficient, and Scalable Confidence-Based-UnderSampling
KRT: kidney replacement therapy
ML: machine learning
PROBAST+AI: Updated Quality, Risk of Bias, and Applicability Assessment Tool for Prediction Models Using Regression or Artificial Intelligence Methods
REDCap: Research Electronic Data Capture
SMOTE: Synthetic Minority Over-Sampling Technique
TRIPOD+AI: Transparent Reporting of a multivariable prediction model for Individual Prognosis or Diagnosis + Artificial Intelligence
XGBoost: extreme gradient boosting

## References

[1] Collins GS, Dhiman P, Ma J (2024). Evaluation of clinical prediction models (part 1): from development to external validation. BMJ.

[2] Steyerberg EW (2019). Clinical Prediction Models: A Practical Approach to Development, Validation, and Updating.

[3] van Smeden M, Reitsma JB, Riley RD, Collins GS, Moons KG (2021). Clinical prediction models: diagnosis versus prognosis. J Clin Epidemiol.

[4] Adhikari S, Normand SL, Bloom J, Shahian D, Rose S (2021). Revisiting performance metrics for prediction with rare outcomes. Stat Methods Med Res.

[5] Cabot JH, Ross EG (2023). Evaluating prediction model performance. Surgery.

[6] Collins GS, Moons KGM, Dhiman P (2024). TRIPOD+AI statement: updated guidance for reporting clinical prediction models that use regression or machine learning methods. BMJ.

[7] Cartus AR, Samuels EA, Cerdá M, Marshall BDL (2023). Outcome class imbalance and rare events: an underappreciated complication for overdose risk prediction modeling. Addiction.

[8] de Paiva BBM, Pereira PD, de Andrade CMV (2023). Potential and limitations of machine meta-learning (ensemble) methods for predicting COVID-19 mortality in a large inhospital Brazilian dataset. Sci Rep.

[9] Liu S, Roemer F, Ge Y (2023). Comparison of evaluation metrics of deep learning for imbalanced imaging data in osteoarthritis studies. Osteoarthr Cartil.

[10] D’Agostino RB, Vasan RS, Pencina MJ (2008). General cardiovascular risk profile for use in primary care: the Framingham Heart Study. Circulation.

[11] Hosmer DW, Lemeshow S, Sturdivant RX Applied Logistic Regression.

[12] Iragorri N, Spackman E (2018). Assessing the value of screening tools: reviewing the challenges and opportunities of cost-effectiveness analysis. Public Health Rev.

[13] Arnett DK, Blumenthal RS, Albert MA (2019). 2019 ACC/AHA guideline on the primary prevention of cardiovascular disease: a report of the American College of Cardiology/American Heart Association task force on clinical practice guidelines. Circulation.

[14] Krist AH, Davidson KW, US Preventive Services Task Force (2020). Behavioral counseling interventions to promote a healthy diet and physical activity for cardiovascular disease prevention in adults with cardiovascular risk factors: US Preventive Services Task Force recommendation statement. JAMA.

[15] Rufibach K (2010). Use of Brier score to assess binary predictions. J Clin Epidemiol.

[16] Moons KGM, Damen JAA, Kaul T (2025). PROBAST+AI: an updated quality, risk of bias, and applicability assessment tool for prediction models using regression or artificial intelligence methods. BMJ.

[17] Hageman S, Pennells L, Ojeda F (2021). SCORE2 risk prediction algorithms: new models to estimate 10-year risk of cardiovascular disease in Europe. Eur Heart J.

[18] Van Calster B, McLernon DJ, van Smeden M, Wynants L, Steyerberg EW, Topic Group ‘Evaluating diagnostic tests and prediction models’ of the STRATOS initiative (2019). Calibration: the Achilles heel of predictive analytics. BMC Med.

[19] Liu T, Krentz A, Lu L, Curcin V (2025). Machine learning based prediction models for cardiovascular disease risk using electronic health records data: systematic review and meta-analysis. Eur Heart J Digit Health.

[20] Liu W, Laranjo L, Klimis H (2023). Machine-learning versus traditional approaches for atherosclerotic cardiovascular risk prognostication in primary prevention cohorts: a systematic review and meta-analysis. Eur Heart J Qual Care Clin Outcomes.

[21] Andersen ES, Birk-Korch JB, Hansen RS (2024). Monitoring performance of clinical artificial intelligence in health care: a scoping review. JBI Evid Synth.

[22] Oettl FC, Pareek A, Winkler PW (2024). A practical guide to the implementation of AI in orthopaedic research, part 6: how to evaluate the performance of AI research?. J Exp Orthop.

[23] Hicks SA, Strümke I, Thambawita V (2022). On evaluation metrics for medical applications of artificial intelligence. Sci Rep.

[24] Megahed FM, Chen YJ, Megahed A, Ong Y, Altman N, Krzywinski M (2021). The class imbalance problem. Nat Methods.

[25] Lever J, Krzywinski M, Altman N (2016). Classification evaluation. Nat Methods.

[26] Kelly CJ, Karthikesalingam A, Suleyman M, Corrado G, King D (2019). Key challenges for delivering clinical impact with artificial intelligence. BMC Med.

[27] Kocak B, Klontzas ME, Stanzione A (2025). Evaluation metrics in medical imaging AI: fundamentals, pitfalls, misapplications, and recommendations. Eur J Radiol Artif Intell.

[28] Carriero A, Luijken K, de Hond A, Moons KGM, van Calster B, van Smeden M (2025). The harms of class imbalance corrections for machine learning based prediction models: a simulation study. Stat Med.

[29] (2021). Recommendations for national SARS-cov-2 testing strategies and diagnostic capacities: interim guidance, 25 June 2021. World Health Organization.

[30] Marcolino MS, Ziegelmann PK, Souza-Silva MVR (2021). Clinical characteristics and outcomes of patients hospitalized with COVID-19 in Brazil: results from the Brazilian COVID-19 registry. Int J Infect Dis.

[31] Yang L, Li J, Wei W (2022). Kidney health in the COVID-19 pandemic: an umbrella review of meta-analyses and systematic reviews. Front Public Health.

[32] Harris PA, Taylor R, Thielke R, Payne J, Gonzalez N, Conde JG (2009). Research electronic data capture (REDCap)--a metadata-driven methodology and workflow process for providing translational research informatics support. J Biomed Inform.

[33] Harris PA, Taylor R, Minor BL (2019). The REDCap consortium: building an international community of software platform partners. J Biomed Inform.

[34] Soriano Marcolino M, Minelli Figueira R, Pereira Afonso Dos Santos J, Silva Cardoso C, Luiz Ribeiro A, Alkmim MB (2016). The experience of a sustainable large scale Brazilian telehealth network. Telemed J E Health.

[35] Bicalho MAC, Aliberti MJR, Delfino-Pereira P (2024). Clinical characteristics and outcomes of COVID-19 patients with preexisting dementia: a large multicenter propensity-matched Brazilian cohort study. BMC Geriatr.

[36] Chen T, Guestrin C XGBoost: a scalable tree boosting system.

[37] Shwartz-Ziv R, Armon A (2022). Tabular data: deep learning is not all you need. Inf Fusion.

[38] Wang L, Wang X, Chen A, Jin X, Che H (2020). Prediction of type 2 diabetes risk and its effect evaluation based on the XGBoost model. Healthcare (Basel).

[39] Wilimitis D, Walsh CG (2023). Practical considerations and applied examples of cross-validation for model development and evaluation in health care: tutorial. JMIR AI.

[40] Bradshaw TJ, Huemann Z, Hu J, Rahmim A (2023). A guide to cross-validation for artificial intelligence in medical imaging. Radiol Artif Intell.

[41] Kohavi R A study of cross-validation and bootstrap for accuracy estimation and model selection.

[42] Adin A, Krainski ET, Lenzi A, Liu Z, Martínez-Minaya J, Rue H (2024). Automatic cross-validation in structured models: is it time to leave out leave-one-out?. Spat Stat.

[43] Lemaitre G, Nogueira F, Aridas CK (2016). Imbalanced-learn: a Python toolbox to tackle the curse of imbalanced datasets in machine learning. arXiv.

[44] Garcia EA, He H, Bai Y, Li S ADASYN: adaptive synthetic sampling approach for imbalanced learning.

[45] Chawla NV, Bowyer KW, Hall LO, Kegelmeyer WP (2002). SMOTE: Synthetic Minority Over-sampling Technique. J Artif Intell Res.

[46] Han H, Wang WY, Mao BH, Huang DS, Zhang XP, Huang GB (2005). Advances in Intelligent Computing.

[47] Nguyen HM, Cooper EW, Kamei K (2011). Borderline over-sampling for imbalanced data classification. Int J Knowledge Eng Soft Data Paradigms.

[48] Douzas G, Bacao F, Last F (2018). Improving imbalanced learning through a heuristic oversampling method based on k-means and SMOTE. Inf Sci (Ny).

[49] More A (2016). Survey of resampling techniques for improving classification performance in unbalanced datasets. arXiv.

[50] Murphy KP (2012). Machine Learning: A Probabilistic Perspective.

[51] Powers DMW (2020). Evaluation: from precision, recall and F-measure to ROC, informedness, markedness and correlation. J Mach Learn Technol.

[52] Stekhoven DJ, Bühlmann P (2012). MissForest--non-parametric missing value imputation for mixed-type data. Bioinformatics.

[53] Sokolova M, Lapalme G (2009). A systematic analysis of performance measures for classification tasks. Inf Process Manag.

[54] Davis J, Goadrich M The relationship between precision-recall and ROC curves.

[55] Vickers AJ, Elkin EB (2006). Decision curve analysis: a novel method for evaluating prediction models. Med Decis Making.

[56] Lipton ZC, Elkan C, Naryanaswamy B (2014). Optimal thresholding of classifiers to maximize F1 measure. Mach Learn Knowl Discov Databases.

[57] Steyerberg EW, Vickers AJ, Cook NR (2010). Assessing the performance of prediction models: a framework for traditional and novel measures. Epidemiology (Sunnyvale).

[58] Huang Y, Li W, Macheret F, Gabriel RA, Ohno-Machado L (2020). A tutorial on calibration measurements and calibration models for clinical prediction models. J Am Med Inform Assoc.

[59] Alba AC, Agoritsas T, Walsh M (2017). Discrimination and calibration of clinical prediction models: users’ guides to the medical literature. JAMA.

[60] Viering T, Loog M (2023). The shape of learning curves: a review. IEEE Trans Pattern Anal Mach Intell.

[61] Simon GJ, Aliferis C (2024). Artificial Intelligence and Machine Learning in Health Care and Medical Sciences: Best Practices and Pitfalls.

[62] Ke JXC, DhakshinaMurthy A, George RB, Branco P (2024). The effect of resampling techniques on the performances of machine learning clinical risk prediction models in the setting of severe class imbalance: development and internal validation in a retrospective cohort. Discov Artif Intell.

[63] Dinov ID (2018). Data Science and Predictive Analytics: Biomedical and Health Applications Using R.

[64] Welvaars K, Oosterhoff JHF, van den Bekerom MPJ, Doornberg JN, van Haarst EP, OLVG Urology Consortium, and the Machine Learning Consortium (2023). Implications of resampling data to address the class imbalance problem (IRCIP): an evaluation of impact on performance between classification algorithms in medical data. JAMIA Open.

[65] van den Goorbergh R, van Smeden M, Timmerman D, Van Calster B (2022). The harm of class imbalance corrections for risk prediction models: illustration and simulation using logistic regression. J Am Med Inform Assoc.

[66] Monaghan TF, Rahman SN, Agudelo CW (2021). Foundational statistical principles in medical research: sensitivity, specificity, positive predictive value, and negative predictive value. Medicina (Kaunas).

[67] (2025). Clinical criteria. DynaMed.

[68] Riley RD, Pate A, Dhiman P, Archer L, Martin GP, Collins GS (2023). Clinical prediction models and the multiverse of madness. BMC Med.

[69] Bozkurt C, Aşuroğlu T (2023). Mortality prediction of various cancer patients via relevant feature analysis and machine learning. SN Comput Sci.

