# Supplementary material for: Beyond Area Under the Receiver Operating Characteristic Curve: Evaluating Predictive Performance Metrics Under Class Imbalance in Real-World Clinical Data
Source: JMIR Form Res. 2026 Jun 24;10:e86379. doi: 10.2196/86379 (PMC13293568; doi:10.2196/86379)
Supplement: Multimedia Appendix 1 [file formative-v10-e86379-s001.docx]

Multimedia Appendix 1. Potential predictors for patients undergoing kidney replacement therapy in COVID-19 patients.

| **Variables** | **Scientific evidence** |
| --- | --- |
| **Demographic** |  |
| Age | 1-7 |
| Sex at birth | 7-9 |
| **Comorbidities** |  |
| Cardiovascular system |  |
| Hypertension | 5,7 |
| Coronary artery disease | 7 |
| Heart failure | 7 |
| Atrial fibrillation/flutter | 7 |
| Ischemic stroke | 7 |
| Chagas disease | - |
| Venous thromboembolism | - |
| COPD | 7 |
| Diabetes mellitus | 2,3,7.8 |
| Obesity (BMI>30kg/m2) | 7 |
| Cirrhosis | - |
| Chronic kidney disease | 1,7 |
| Rheumatologic disease / connective tissue | - |
| HIV infection | - |
| Malignant neoplasm | 7 |
| Previous transplantation | - |
| Dementia | - |
| Number of comorbidities | - |
| **Functional status of the patient before COVID-19** | 3 |
| Robust |  |
| Mild frailty |  |
| Moderate frailty |  |
| Severe frailty |  |
| **Medication** |  |
| Oral anticoagulant | - |
| Inhaled corticosteroids | - |
| Oral corticoids | - |
| Imunossupressor | - |
| **Lifestyle** |  |
| Illicit drugs use | - |
| Alcohol abuse | - |
| Current smoking | 7 |
| **Vaccinations** | - |
| Number of vaccine doses |  |
| Which vaccine |  |
| **Clinical findings** |  |
| Sensory impairment | 3 |
| Glasgow coma scale | 3 |
| Systolic blood pressure (mmHg) | 5 |
| Diastolic blood pressure (mmHg) | 5 |
| Use of vasoactive amines | 5,10 |
| Combination of systolic pressure and use of amines | 5 |
| Combination of diastolic pressure and use of amines | 5 |
| Heart rate (bpm^1^) | 5 |
| Respiratory rate (bpm^2^) | - |
| Temperature (ºC) | 5 |
| O2 saturation (%) | 4,5,9 |
| Saturation O2/FiO2 | 4,5 |
| Invasive mechanical ventilation at admission | 3,7,8,10,11 |
| Invasive mechanical ventilation at any time of hospitalization | 3,7,8,10,11 |
| Non-invasive mechanical ventilation | 3 |
| **Laboratory findings** |  |
| Hemoglobin (g/dL) | - |
| Leukocytes (cells/mm3) | 5,9 |
| Neutrophils (cels/mm3) | 6,9 |
| Lymphocytes (cels/mm3) | 5,6 |
| Neutrophil/lymphocyte ratio | 5,6 |
| Platelets (cels/mm3) | 5 |
| D-dimer (ng/ml) | 5 |
| Ferritin (ng/mL) | - |
| Protein C reactive (mg/L) | 1,9,10 |
| Aspartate aminotransferase (U/L) | 5 |
| Alanine aminotransferase (U/L) |  |
| Troponin | - |
| Lactate | - |
| Total Bilirubin (mg/dL) | - |
| Blood urea nitrogen (mg/dL) | 5,9 |
| Creatinine (mg/dL) | 2,5,8-10 |
| PO2/FiO2 ratio | 4,5 |
| pH | - |
| arterial pCO2 | - |
| arterial pO2 | 5 |
| Bicarbonate | 9 |

BMI: Body Mass Index; COPD: Chronic Obstructive Pulmonary Disease; HIV: Human Immunodeficiency Virus; PO2/FiO2 ratio: Arterial Oxygen Partial Pressure to Fraction of Inspired Oxygen Ratio; Saturation O2/FiO2: Oxygen Saturation to Fraction of Inspired Oxygen Ratio.

**References**

1. Diebold M, Schaub S, Landmann E, et al. Acute kidney injury in patients with COVID-19: a retrospective cohort study from Switzerland. *Swiss Med Wkly* 2021;151:w20482.
2. Flechet M, Güiza F, Schetz M, et al. AKI predictor, an online prognostic calculator for acute kidney injury in adult critically ill patients: development, validation and comparison to serum neutrophil gelatinase-associated lipocalin. *IntensiveCare Med* 2021;43(6):764–73.
3. França ARM, Ferreira JC, Valente TM, et al. Development and validation of a machine learning model to predict the use of renal replacement therapy in 14,374 patients with COVID-19. *J Crit Care* 2024;80:154480.
4. See YP, Young BE, Ang LW, et al. Risk factors for development of acute kidney injury in COVID-19 patients: a retrospective observational cohort study. *Nephron* 2021;145(3):256–64.
5. Vaid A, Somani S, Russak AJ, et al. Predictive approaches for acute dialysis requirement and death in COVID-19. *Clin J Am Soc Nephrol* 2021;16(8):1158–68.
6. Wang F, Ran L, Qian C, et al. Epidemiology and outcomes of acute kidney injury in COVID-19 patients with acute respiratory distress syndrome: a multicenter retrospective study. *Blood Purif* 2021;50(4–5):499–505.
7. Yang L, Guo T, Liu D, et al. Kidney health in the COVID-19 pandemic: an umbrella review of meta-analyses and systematic reviews. *Front Public Health* 2022;10:963667.
8. Figueiredo FA, Freitas DF, Camargo MFC, et al. Development and validation of the MMCD score to predict kidney replacement therapy in COVID-19 patients. *BMC Med* 2022;20(1):324.
9. Rodriguez VA, Chang MG, Milinovich A, et al. Development and validation of prediction models for mechanical ventilation, renal replacement therapy, and readmission in COVID-19 patients. J *Am Med Inform Assoc* 2021;28(7):1480–8.
10. Doher MP, Torres de Carvalho FR, Scherer PF, et al. Acute kidney injury and renal replacement therapy in critically ill COVID-19 patients: risk factors and outcomes - a single-center experience in Brazil. *Blood Purif* 2021;50(4–5):520–30.
11. Lumlertgul N, Pirondini L, Cooney E, et al. Acute kidney injury prevalence, progression and long-term outcomes in critically ill patients with COVID-19: a cohort study. *Ann Intensive Care* 2021;11(1):1–11.
