# Supplementary material for: Beyond Area Under the Receiver Operating Characteristic Curve: Evaluating Predictive Performance Metrics Under Class Imbalance in Real-World Clinical Data
Source: JMIR Form Res. 2026 Jun 24;10:e86379. doi: 10.2196/86379 (PMC13293568; doi:10.2196/86379)
Supplement: Multimedia Appendix 2 [file formative-v10-e86379-s002.docx]

Multimedia Appendix 2. Potential predictors for in-hospital mortality in COVID-19 patients.

| **Variables** | **Scientific evidence** |
| --- | --- |
| **Demographic** |  |
| Age | 1-31 |
| **Comorbidities** |  |
| Cardiovascular system |  |
| Hypertension | 3,4,9,12,21,24,28 |
| Coronary artery disease | 5,12,17,23,25,28,32,33 |
| Heart failure | 4,12,28,32,33 |
| Atrial fibrillation/flutter | 4,32 |
| Ischemic stroke | 5,33 |
| Chagas disease | 4,32 |
| Venous thromboembolism | 32 |
| COPD | 9,12,28,32,33 |
| Diabetes mellitus | 3,9,12,17,28,32,33 |
| Obesity (BMI>30kg/m2) | 3,12,17,18,21,32 |
| Cirrhosis | 12,32,33 |
| Chronic kidney disease | 3,12,21,28,32,33 |
| Rheumatologic disease / connective tissue | 32 |
| HIV infection | 12,21,32,33 |
| Malignant neoplasm | 16,21,28,32,33 |
| Previous transplantation | 12,21,32 |
| Dementia | 32,33,34 |
| Number of comorbidities | 8,14,16,19,33,35 |
| **Functional status of the patient before COVID-19** | 36 |
| Robust |  |
| Mild frailty |  |
| Moderate frailty     Severe frailty |  |
| **Medication** |  |
| Oral anticoagulants | 37 |
| Inhaled corticosteroids | 38 |
| Oral corticoids | 39 |
| Imunossupressor | 40,41 |
| **Lifestyle** |  |
| Illicit drugs use | 41 |
| Alcohol abuse | 41,42 |
| Current smoking | 43 |
| Previous smoking | 43 |
| **Vaccinations** | - |
| Number of vaccine doses |  |
| Which vaccine |  |
| **Clinical findings** |  |
| Sensory impairment | 7,14,16 |
| Glasgow coma scale | 10,31 |
| Systolic blood pressure (mmHg) | 7,10,31 |
| Diastolic blood pressure (mmHg) | 2,11 |
| Mean blood pressure (mmHg) | 2,11,31 |
| Use of vasoactive amines | 31 |
| Combination of systolic pressure and use of amines | 31 |
| Combination of diastolic pressure and use of amines | 31 |
| Heart rate (bpm1) | 7,19 |
| Respiratory rate (bpm2) | 7,8,14,18,32 |
| Temperature (ºC) | 7,18,20 |
| O2 saturation (%) | 2,7,9,11,14,21,24,29,32 |
| Saturation O2/FiO2 | 17,19 |
| Invasive mechanical ventilation at admission | 1,4 |
| Invasive mechanical ventilation at any time of hospitalization | - |
| **Laboratory findings** |  |
| Hemoglobin (g/dL) | 7 |
| Leukocytes (cells/mm3) | 7,22,24 |
| Neutrophils (cels/mm3) | 9,15 |
| Lymphocytes (cels/mm3) | 1,13,23,24 |
| Neutrophil/lymphocyte ratio | 16-18,20,27,30 |
| Platelets (cels/mm3) | 8,10,17,19,31 |
| D-dimer (ng/ml) | 13,23,26,27,31 |
| Ferritin (ng/mL) | 26 |
| Protein C reactive (mg/L) | 1,2,4,9,13,14,18,19,21,25,25,27,44 |
| Aspartate aminotransferase (U/L) | 5,20,25,44 |
| Alanine aminotransferase (U/L) | - |
| Troponin | 15,18 |
| Lactate | 35 |
| Bilirubin (mg/dL) | 16,30,31 |
| Blood urea nitrogen (mg/dL) | 2,7,14,19,35,44 |
| Creatinine (mg/dL) | 7-9,11,22,25,31 |
| PO2/FiO2 ratio | 8,31 |
| pH | - |
| arterial pCO2 | - |
| arterial pO2 | 11 |
| Bicarbonate | 44 |

BMI: Body Mass Index; COPD: Chronic Obstructive Pulmonary Disease; HIV: Human Immunodeficiency Virus; PO2/FiO2 ratio: Arterial Oxygen Partial Pressure to Fraction of Inspired Oxygen Ratio; Saturation O2/FiO2: Oxygen Saturation to Fraction of Inspired Oxygen Ratio.

References

1. Allenbach Y, Saadoun D, Maalouf G, et al. Development of a multivariate prediction model of intensive care unit transfer or death: a French prospective cohort study of hospitalized COVID-19 patients. *PLoS One* 2020;15(10):e0240711.
2. Altschul DJ, Unda SR, Benton J, et al. A novel severity score to predict inpatient mortality in COVID-19 patients. *Sci Rep* 2020;10(1):16726.
3. Bello-Chavolla OY, Bahena-López JP, Antonio-Villa NE, et al. Predicting mortality due to SARS-CoV-2: a mechanistic score relating obesity and diabetes to COVID-19 outcomes in Mexico. *J Clin Endocrinol Metab*. 2020;105(8):2752–61.
4. Cena T, Arduini S, Righi E, et al. Predictors of intubation and mortality in COVID-19 patients: a retrospective study. *J Anesth Analg Crit Care* 2021;1(1):19.
5. Chen R, Liang W, Jiang M, et al. Risk factors of fatal outcome in hospitalized subjects with coronavirus disease 2019 from a nationwide analysis in China. *Chest* 2020;158(1):97–105.
6. Das AK, Mishra S, Gopalan SS. Predicting COVID-19 community mortality risk using machine learning and development of an online prognostic tool. *PeerJ* 2020;8:e10083.
7. Faisal M, Mohammed M, Richardson D, et al. Development and validation of automated computer-aided risk scores to predict in-hospital mortality for emergency medical admissions with COVID-19: a retrospective cohort development and validation study. *BMJ Open* 2022;12(8):e050274.
8. Fumagalli C, Rozzini R, Vannini M, et al. Clinical risk score to predict in-hospital mortality in COVID-19 patients: a retrospective cohort study. *BMJ Open* 2020;10(9):e040729.
9. Galloway JB, Norton S, Barker RD, et al. A clinical risk score to identify patients with COVID-19 at high risk of critical care admission or death: an observational cohort study. *J Infect* 2020;81(2):282–8.
10. Gue YX, Tennyson M, Gao J, et al. Development of a novel risk score to predict mortality in patients admitted to hospital with COVID-19. *Sci Rep* 2020;10(1):21379.
11. Hajifathalian K, Kumar S, Newberry C, et al. Development and external validation of a prediction risk model for short-term mortality among hospitalized US COVID-19 patients: a proposal for the COVID-AID risk tool. *PLoS One* 2020;15(9):e0239536.
12. Halalau A, Imam Z, Karabon P, et al. External validation of a clinical risk score to predict hospital admission and in-hospital mortality in COVID-19 patients. *Ann Med* 2021;53(1):78–86.
13. Hu C, Liu Z, Jiang Y, et al. Early prediction of mortality risk among patients with severe COVID-19, using machine learning. *Int J Epidemiol* 2021;49(6):1918–29.
14. Knight SR, Ho A, Pius R, et al. Risk stratification of patients admitted to hospital with COVID-19 using the ISARIC WHO Clinical Characterisation Protocol: development and validation of the 4C Mortality Score. *BMJ* 2020;370:m3339.
15. Li P, Chen L, Liu Z, et al. Clinical features and short-term outcomes of elderly patients with COVID-19. *Int J Infect Dis* 2020;97:245–50.
16. Liang W, Liang H, Ou L, et al. Development and validation of a clinical risk score to predict the occurrence of critical illness in hospitalized patients with COVID-19. *JAMA Intern Med* 2020;180(8):1081–9.
17. Nicholson CJ, Wooster L, Sigurslid HH, et al. Estimating risk of mechanical ventilation and in-hospital mortality among adult COVID-19 patients admitted to Mass General Brigham: the VICE and DICE scores. *EClinicalMedicine* 2021;33:100765.
18. Garibaldi BT, Fiksel J, Muschelli J, et al. Patient trajectories among persons hospitalized for COVID-19: a cohort study. *Ann Intern Med* 2021;174(1):33–41.
19. Marcolino MS, Ziegelmann PK, Souza-Silva MV, et al. ABC2-SPH risk score for in-hospital mortality in COVID-19 patients: development, external validation and comparison with other available scores. *Int J Infect Dis* 2021;110:281–308.
20. Mei Q, Wang F, Yang Y, et al. Development and validation of prognostic model for predicting mortality of COVID-19 patients in Wuhan, China. *Sci Rep* 2020;10(1):22451.
21. Nunez-Gil IJ, Estrada V, Fernandez-Perez C, et al. Mortality risk assessment in Spain and Italy, insights of the HOPE COVID-19 registry. *Intern Emerg Med* 2020;15(8):1459–71.
22. Pascual Gómez NF, Fernández Rozas S, Bonilla Galicia JA, et al. Potenciales biomarcadores predictores de mortalidad en pacientes COVID-19 en el Servicio de Urgencias. *Rev Esp Quimioter* 2020;33(4):267–73.
23. Shang Y, Liu T, Wei Y, et al. Scoring systems for predicting mortality for severe patients with COVID-19. *EClinicalMedicine* 2020;24:100426.
24. Soto-Mota A, Marfil-Garza BA, Cedillo-Ramirez L, et al. The low-harm score for predicting mortality in patients diagnosed with COVID-19: a multicentric validation study. *Am Coll Emerg Physicians Open* 2020;1(6):1436–43.
25. Sourij H, Aziz F, Brägelmann J, et al. COVID-19 fatality prediction in people with diabetes and prediabetes using a simple score upon hospital admission. D*iabetes Obes Metab* 2020;23(2):589–98.
26. Wang J, Jiang M, Chen X, et al. Thrombo-inflammatory features predicting mortality in patients with COVID-19: the FAD-85 score. *J Int Med Res* 2020;48(9):300060520949175.
27. Weng Z, Chen Q, Li S, et al. ANDC: an early warning score to predict mortality risk for patients with Coronavirus Disease 2019. *J Transl Med* 2020;18:328.
28. Williams RD, Alsharif MN, Mi E, et al. Seek COVER: using a disease proxy to rapidly develop and validate a personalized risk calculator for COVID-19 outcomes in an international network. *BMC Med Res Methodol* 2022;22(1):35.
29. Yadaw AS, Li YC, Bose S, et al. Clinical features of COVID-19 mortality: development and validation of a clinical prediction model. *Lancet Digit Health* 2020;2(10):e516–25.
30. Zhang S, Guo M, Duan L, et al. Development and validation of a risk factor-based system to predict short-term survival in adult hospitalized patients with COVID-19: a multicenter, retrospective, cohort study. *Crit Care* 2020;24(1):438.
31. Zhou F, Yu T, Du R, et al. Clinical course and risk factors for mortality of adult inpatients with COVID-19 in Wuhan, China: a retrospective cohort study. *Lancet* 2020;395(10229):1054–62.
32. Gavelli F, Patrucco F, Vancheri F, et al. Clinical stability and in-hospital mortality prediction in COVID-19 patients presenting to the Emergency Department. *Minerva Med* 2021;112(1):118–23.
33. Tuty Kuswardhani RA, Henrina J, Pranata R, et al. Charlson comorbidity index and a composite of poor outcomes in COVID-19 patients: a systematic review and meta-analysis. *Diabetes Metab Syndr* 2020;14(6):2103–9.
34. Secnik J, Hedberg P, Josephson M, et al. Hospitalisation for COVID-19 is associated with excess mortality in older patients treated in primary care before hospitalisation. *BMC Geriatr* 2022;22(1):355.
35. Guan WJ, Liang WH, Zhao Y, et al. Comorbidity and its impact on 1590 patients with COVID-19 in China: a nationwide analysis. *Eur Respir J* 2020;55(5):2000547.
36. Du RH, Liang LR, Yang CQ, et al. Predictors of mortality for patients with COVID-19 pneumonia caused by SARS-CoV-2: a prospective cohort study. *Eur Respir J* 2020;55(5):2000524.
37. Ioannou GN, Locke E, Green P, et al. Risk factors for hospitalization, mechanical ventilation, or death among 10 131 US veterans with SARS-CoV-2 infection. *JAMA Netw Open* 2020;3(9):e2022310.
38. Ng WH, Tipih T, Makoah NA, et al. Comorbidities in SARS-CoV-2 patients: a systematic review and meta-analysis. *mBio* 2021;12(1):e03647–20.
39. Ji D, Zhang D, Xu J, et al. Prediction for progression risk in patients with COVID-19 pneumonia: the CALL score. *Clin Infect Dis* 2020;71(6):1393–9.
40. Zou X, Li S, Fang M, et al. Acute physiology and chronic health evaluation II score as a predictor of hospital mortality in patients of coronavirus disease 2019. *Crit Care Med* 2020;48(8):e657–65.
41. Shi Y, Yu X, Zhao H, et al. Host susceptibility to severe COVID-19 and establishment of a host risk score: findings of 487 cases outside Wuhan. *Crit Care* 2020;24(1):108.
42. Vaid A, Somani S, Russak AJ, et al. Machine learning to predict mortality and critical events in COVID-19 positive New York City patients. *J Med Internet Res* 2020;22(11):e24018.
43. Sousa GJB, Garces TS, Cestari VRF, et al. Estimation and prediction of COVID-19 cases in Brazilian metropolises. *Rev Lat Am Enfermagem* 2020 Jun 26;28:e3345.
44. Gupta RK, Marks M, Samuels THA, et al. Systematic evaluation and external validation of 22 prognostic models among hospitalized adults with COVID-19: an observational cohort study. *Eur Respir J* 2020;56(6):2003498.
