# Supplementary material for: Beyond Area Under the Receiver Operating Characteristic Curve: Evaluating Predictive Performance Metrics Under Class Imbalance in Real-World Clinical Data
Source: JMIR Form Res. 2026 Jun 24;10:e86379. doi: 10.2196/86379 (PMC13293568; doi:10.2196/86379)
Supplement: Multimedia Appendix 3 [file formative-v10-e86379-s003.docx]

Multimedia Appendix 3. Final proportions in the training partition after application of each rebalancing strategy.

| **Sampling method** | **Death** | **KRT** |
| --- | --- | --- |
| **Unbalanced** | 1:4.6 | 1:9.5 |
| **RUS** | 1:1 | 1:1 |
| **UBR** | 1:1.8 | 1:4.3 |
| **e2sc_us** | 1:1.8 | 1:4.3 |
| **CNN** | 1:1.5 | 1:2.3 |
| **NM1** | 1:1 | 1:1 |
| **NM2** | 1:1 | 1:1 |
| **ROS** | 1:1 | 1:1 |
| **ADASYN** | 1:1 | 1:1 |
| **SMOTE** | 1:1 | 1:1 |
| **BorderlineSMOTE** | 1:1 | 1:1 |
| **SVMSMOTE** | 1:1 | 1:1 |
| **KMeansSMOTE** | 1:1 | 1:1 |

ADASYN: adaptive synthetic, BorderlineSMOTE: borderline synthetic minority oversampling technique, CNN: condensed nearest neighbour, e2sc_us: effective, efficient, and scalable confidence-based undersampling, KMeansSMOTE: K-means synthetic minority oversampling technique, KRT: kidney replacement therapy, NM1: near miss 1, NM2: near miss 2, ROS: random oversampling, RUS: random undersampling, SMOTE: the synthetic minority over-sampling technique, SVMSMOTE: support vector machine synthetic minority oversampling technique, UBR: redundancy-based undersampling.
