# Supplementary material for: Beyond Area Under the Receiver Operating Characteristic Curve: Evaluating Predictive Performance Metrics Under Class Imbalance in Real-World Clinical Data
Source: JMIR Form Res. 2026 Jun 24;10:e86379. doi: 10.2196/86379 (PMC13293568; doi:10.2196/86379)
Supplement: Multimedia Appendix 4 [file formative-v10-e86379-s004.docx]

Multimedia Appendix 4. Hyperparameters evaluated for optimization using XGBoost.

| Name | Values |
| --- | --- |
| booster | **gbtree**, gblinear, dart |
| eta (learning_rate) | 0.1, 0.2, **0.3,** 0.4, 0.5, 0.6, 0.7, 0.8, 0.9 |
| max_depth | 2, 4, **6**, 8, 10 |

The default values ​​defined by the developers are indicated in bold.
