# Supplementary material for: Beyond Area Under the Receiver Operating Characteristic Curve: Evaluating Predictive Performance Metrics Under Class Imbalance in Real-World Clinical Data
Source: JMIR Form Res. 2026 Jun 24;10:e86379. doi: 10.2196/86379 (PMC13293568; doi:10.2196/86379)
Supplement: Multimedia Appendix 5 [file formative-v10-e86379-s005.docx]

Multimedia Appendix 5. Definitions and characteristics of the metrics frequently used to evaluate performance in predictive models, using the machine learning and the statistics terminology.

| **Metric** |  | | **Definition** | | **Benefits** | | **Limitations** | |
| --- | --- | --- | --- | --- | --- | --- | --- | --- |
| **ML term** | **Statistics term** | |  | |  | |  | |
| **Confusion matrix** | **Confusion matrix** | | A table that summarizes the number of correct and incorrect predictions for each class. | | It provides a detailed breakdown of a model’s performance, is useful for diagnosing the types of errors the model makes and serves as a basis for calculating other metrics. | | It can be overwhelming for large datasets or those with many classes. | |
| **Accuracy** | | **Accuracy** | The proportion of true results (both TP and TN) out of the total number of cases evaluated. | It is easy to understand and interpret and serves as a good measure when the target classes are balanced. | | It can be misleading when classes are imbalanced, as it does not distinguish between different types of classification errors. | |  |
| **AUROC** | | **AUROC** | It indicates the probability that a classifier will correctly rank a randomly selected positive instance higher than a randomly selected negative one. | It is a good measure of a model’s discriminative power, providing a quantitative summary of the ROC curve. It allows for the comparison of different models using a single value and is independent of the decision threshold. | | It does not capture class-specific information and can be overly optimistic when applied to imbalanced datasets. | |  |
| **Precision for positive class** | | **Positive predictive value** | The proportion of TP among all positive predictions made by the model. | It is particularly important when the cost of FP is high. | | It does not take FN into account and can be misleading when the classes are imbalanced. | |  |
| **Recall for positive class** | | **Sensibility** | The proportion of cases correctly predicted as positive. | It is important when it is essential to capture as many positive instances as possible. | | It does not take FP into account and can be misleading when the classes are imbalanced. | |  |
| **Precision for negative class** | | **Negative predictive value** | The proportion of TN among all negative predictions made by the model. | It is particularly important when the cost of FN is high. | | It does not take FP into account and can be misleading when the classes are imbalanced. | |  |
| **Recall for negative class** | | **Specificity** | The proportion of cases correctly predicted as negative. | It is important when it is essential to capture as many negative instances as possible. | | It does not take FN into account and can be misleading when the classes are imbalanced. | |  |
| **F1 Score** | | **F1 Score** | The harmonic mean of precision and recall. | It balances precision and recall, reducing the impact of large differences between them and preventing the model from excessively favoring one of the metrics. It can be calculated for each class. | | It may not be suitable when you want to emphasize either precision or recall. | |  |
| **Macro-F1** | | **Macro-F1** | The arithmetic mean of the F1 scores across the classes. | It is good for evaluating overall model performance, especially useful when the classes are imbalanced. As a single statistic, it is more robust to class imbalance. | | It may not be suitable when you want to emphasize the performance of a specific class, as it does not account for per-class precision and recall variations. | |  |

AUROC: area under the receiving operator characteristic curve; FN: false negative; FP: false positive; ML: machine learning; TN: true negative; TP: true positive.

# References

1. Adhikari S, Normand SL, Bloom J, et al. Revisiting performance metrics for prediction with rare outcomes. *Stat Methods Med Res* 2021;30(9):2352–66.
2. Collins GS, Moons KGM, Dhiman P, et al. TRIPOD+AI statement: updated guidance for reporting clinical prediction models that use regression or machine learning methods. *BMJ* 2024;385:e078378.
3. Davis J, Goadrich M. The relationship between precision-recall and ROC curves. In: Proc 23rd Int Conf Mach Learn (ICML) 2006.
4. Lipton ZC, Elkan C, Naryanaswamy B. Machine learning and knowledge discovery in databases. In: ECML PKDD 2014. *Lect Notes Comput Sci* 2014;8725:225–39.
5. Powers DMW. Evaluation: from precision, recall and F-measure to ROC, informedness, markedness and correlation. *Int J Mach Learn Technol* 2011;2(1):37–63.
6. Sokolova M, Lapalme G. A systematic analysis of performance measures for classification tasks. *Inf Process Manag* 2009;45(4):427–37.
7. Steyerberg EW. Clinical prediction models: a practical approach to development, validation, and updating. 2nd ed. Cham: Springer Nature Switzerland AG; 2019.
