# Supplementary material for: Beyond Area Under the Receiver Operating Characteristic Curve: Evaluating Predictive Performance Metrics Under Class Imbalance in Real-World Clinical Data
Source: JMIR Form Res. 2026 Jun 24;10:e86379. doi: 10.2196/86379 (PMC13293568; doi:10.2196/86379)
Supplement: Multimedia Appendix 6 [file formative-v10-e86379-s006.docx]

TP: true positive; TN: true negative; FP: false positive; FN: false negative.

$$\mathbf{=0.667}$$

$$\mathbf{=0.882}$$

**POSITIVE**

**POSITIVE**

**NEGATIVE**

**NEGATIVE**

**PREDICTED**

**ACTUAL**

**TRUE Positive**

**FALSE Negative**

**FALSE Positive**

**TRUE Negative**


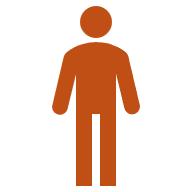

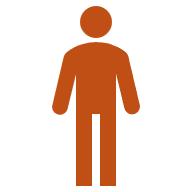

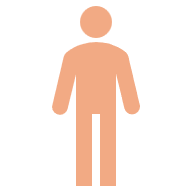

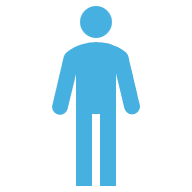

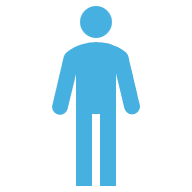

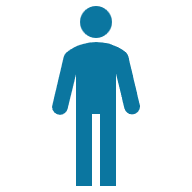

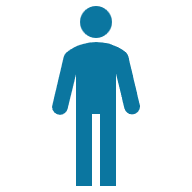

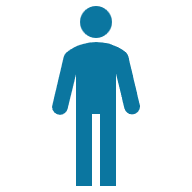

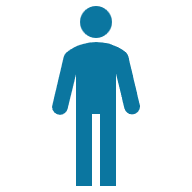

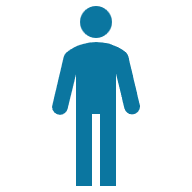

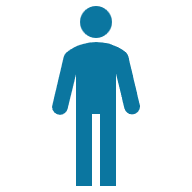

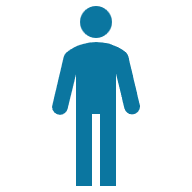

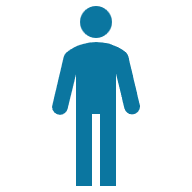

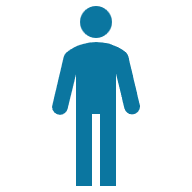

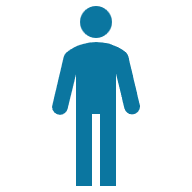

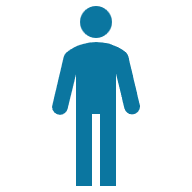

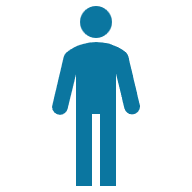

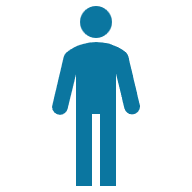

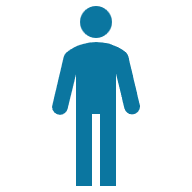

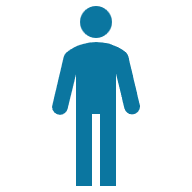

$$\boldsymbol{Acuraccy}\mathbf{=}\frac{\boldsymbol{TP}\mathbf{+}\boldsymbol{TN}}{\boldsymbol{TP}\mathbf{+}\boldsymbol{TN}\mathbf{+}\boldsymbol{FP}\mathbf{+}\boldsymbol{FN}}$$

$$\mathbf{=}\frac{\boldsymbol{2+15}}{\mathbf{2+15+2+1}}$$

$$\mathbf{=0.850}$$

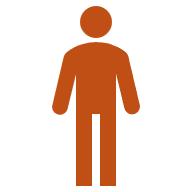

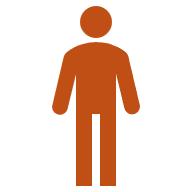

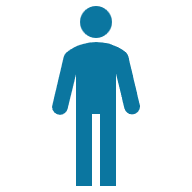

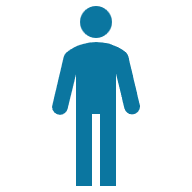

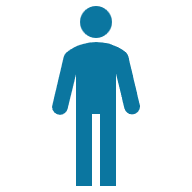

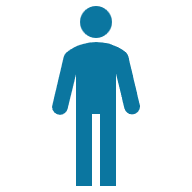

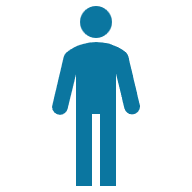

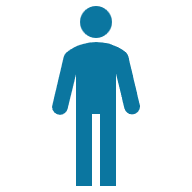

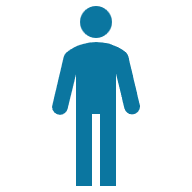

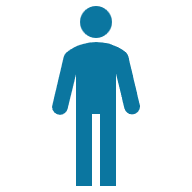

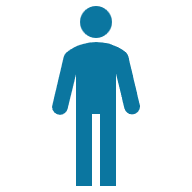

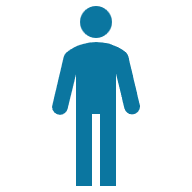

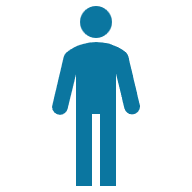

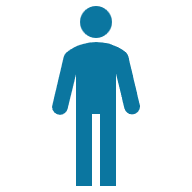

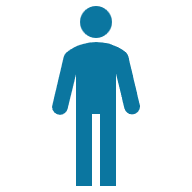

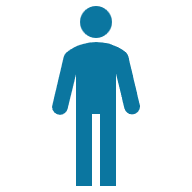

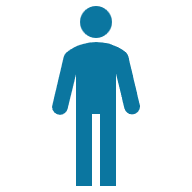

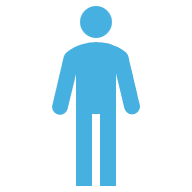

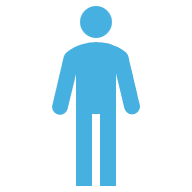

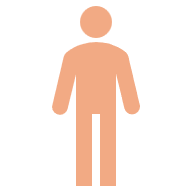

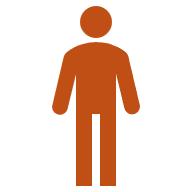

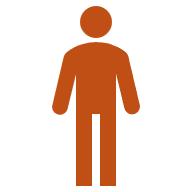

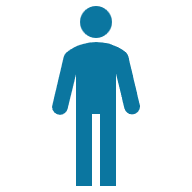

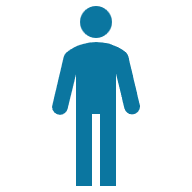

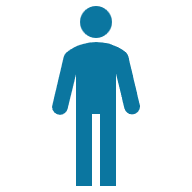

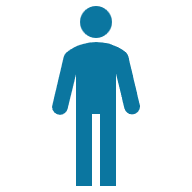

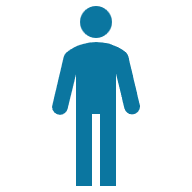

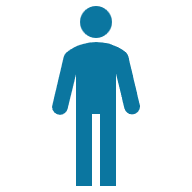

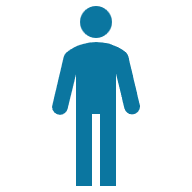

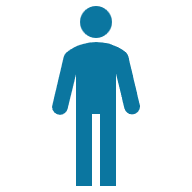

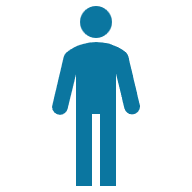

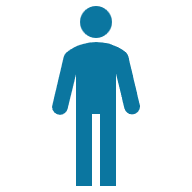

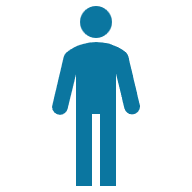

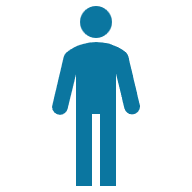

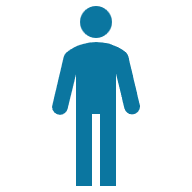

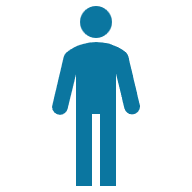

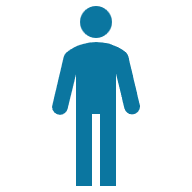

$$\boldsymbol{Positive predicted value}\mathbf{=}\frac{\boldsymbol{TP}}{\boldsymbol{TP}\mathbf{+}\boldsymbol{FN}}$$

$$\mathbf{=}\frac{\boldsymbol{2}}{\mathbf{2+2}}$$

$$\mathbf{=0.500}$$

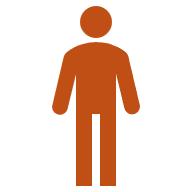

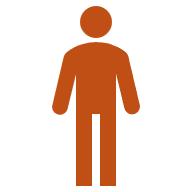

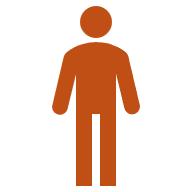

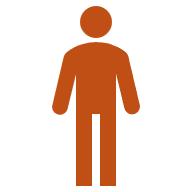

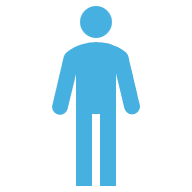

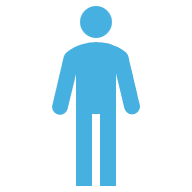

$$\mathbf{=}\frac{\boldsymbol{2}}{\mathbf{2+1}}$$

$$\boldsymbol{Sensivity}\mathbf{=}\frac{\boldsymbol{TP}}{\boldsymbol{TP}\mathbf{+}\boldsymbol{FN}}$$

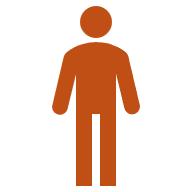

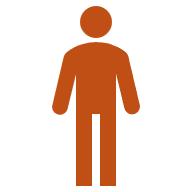

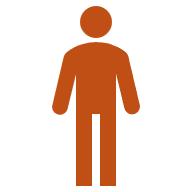

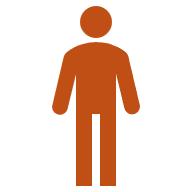

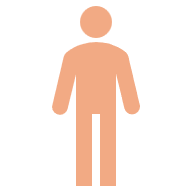

$$\boldsymbol{Negative predited value}\mathbf{=}\frac{\boldsymbol{TN}}{\boldsymbol{TN}\mathbf{+}\boldsymbol{FN}}$$

$$\mathbf{=}\frac{\boldsymbol{15}}{\mathbf{15+1}}$$

$$\mathbf{=0.938}$$

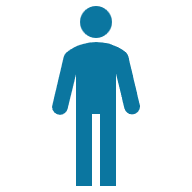

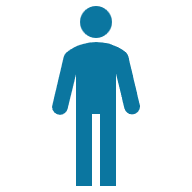

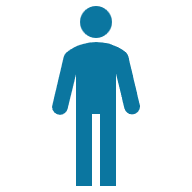

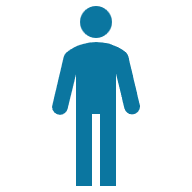

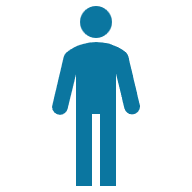

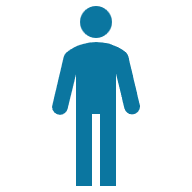

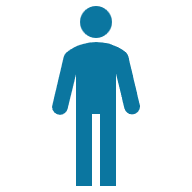

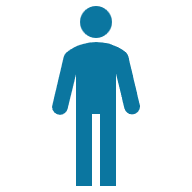

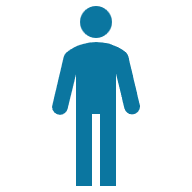

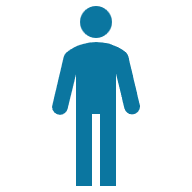

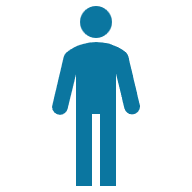

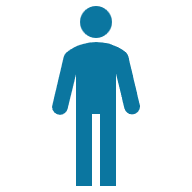

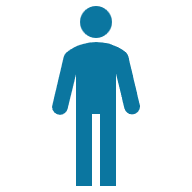

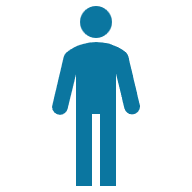

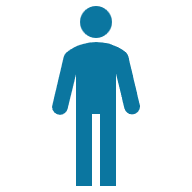

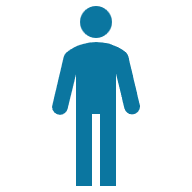

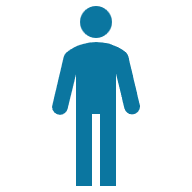

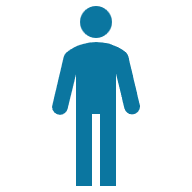

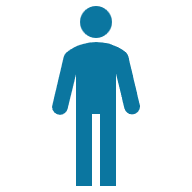

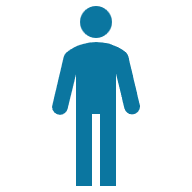

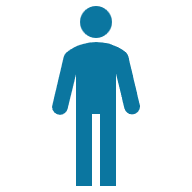

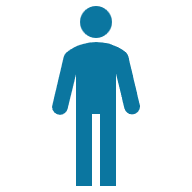

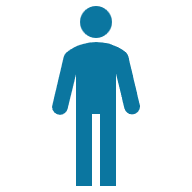

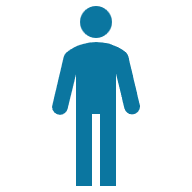

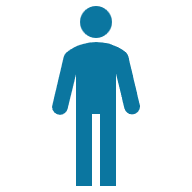

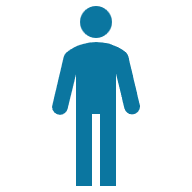

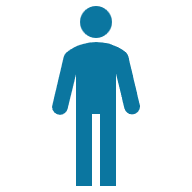

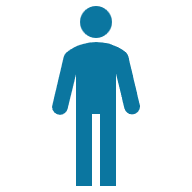

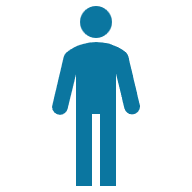

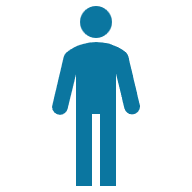

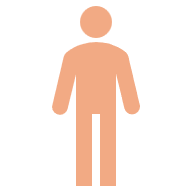

$$\boldsymbol{Specifity}\mathbf{=}\frac{\boldsymbol{TN}}{\boldsymbol{TN}\mathbf{+}\boldsymbol{FP}}$$

$$\mathbf{=}\frac{\boldsymbol{15}}{\mathbf{15+2}}$$

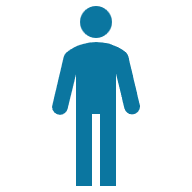


TP: true positive; TN: true negative; FP: false positive; FN: false negative.

**POSITIVE CLASS**

$$\boldsymbol{F}\boldsymbol{1}\mathbf{=2*}\frac{\boldsymbol{Precision*Recall}}{\boldsymbol{Precision+ Recall}}$$

$$\mathbf{=2*}\frac{\boldsymbol{0.500*0.667}}{\boldsymbol{0.500}\mathbf{+}\boldsymbol{0.667}}$$

$$\mathbf{=0.572}$$

**NEGATIVE CLASS**

$$\boldsymbol{F}\boldsymbol{1}\mathbf{=2*}\frac{\boldsymbol{Precision*Recall}}{\boldsymbol{Precision+Recall}}$$

$$\mathbf{=2*}\frac{\boldsymbol{0.882*0.938}}{\boldsymbol{0.882}\mathbf{+}\boldsymbol{0.938}}$$

$$\mathbf{=0.909}$$

$$\boldsymbol{Macro F}\boldsymbol{1}\mathbf{=}\frac{\boldsymbol{F}\boldsymbol{1 positive class+F}\boldsymbol{1 negative class}}{\boldsymbol{2}}$$

$$\mathbf{=}\frac{\boldsymbol{0.572+ 0.909}}{\boldsymbol{2}}$$

$$\mathbf{=0.740}$$

**POSITIVE**

**POSITIVE**

**NEGATIVE**

**NEGATIVE**

**PREDICTED**

**ACTUAL**

**TRUE Positive**

**FALSE Negative**

**FALSE Positive**

**TRUE Negative**

$$\boldsymbol{Recall ou sensivity}\mathbf{=}\frac{\boldsymbol{TP}}{\boldsymbol{TP}\mathbf{+}\boldsymbol{FN}}$$

$$\mathbf{=}\frac{\boldsymbol{2}}{\boldsymbol{2}\mathbf{+}\boldsymbol{1}}$$

$$\mathbf{=0.667}$$

$$\boldsymbol{Precision or positive predicted value}\mathbf{=}\frac{\boldsymbol{TP}}{\boldsymbol{TP}\mathbf{+}\boldsymbol{FP}}$$

$$\mathbf{=}\frac{\boldsymbol{2}}{\mathbf{2+2}}$$

$$\mathbf{=0.500}$$

$$\boldsymbol{Precision or negative predited value}\mathbf{=}\frac{\boldsymbol{TN}}{\boldsymbol{TN}\mathbf{+}\boldsymbol{FN}}$$

$$\mathbf{=}\frac{\boldsymbol{14}}{\boldsymbol{14}\mathbf{+1}}$$

$$\mathbf{=0.938}$$

$$\boldsymbol{Recall or specifity}\mathbf{=}\frac{\boldsymbol{TN}}{\boldsymbol{TN}\mathbf{+}\boldsymbol{FP}}$$

$$\mathbf{=}\frac{\boldsymbol{15}}{\boldsymbol{15}\mathbf{+}\boldsymbol{2}}$$

$$\mathbf{=0.882}$$

**References**

1. Adhikari S, Normand SL, Bloom J, et al. Revisiting performance metrics for prediction with rare outcomes. *Stat Methods Med Res* 2021;30(9):2352–66.
2. Davis J, Goadrich M. The relationship between precision-recall and ROC curves. Proceedings of the 23rd International Conference on Machine Learning - ICML’06. 2006:233–40.
3. Dietterich TG. Approximate statistical tests for comparing supervised classification learning algorithms. *Neural Comput* 1998;10(7):1895–923.
4. Hicks SA, Strømme P, Thambawita V, et al. On evaluation metrics for medical applications of artificial intelligence. *Sci Rep* 2022;12:5979.
5. Hosmer DW, Lemeshow S, Sturdivant RX. Applied logistic regression. 3rd ed. Hoboken: Wiley; 2013.
6. Lever J, Krzywinski M, Altman N. Classification evaluation. *Nat Methods* 2016;13(8):603–4.
7. Sokolova M, Lapalme G. A systematic analysis of performance measures for classification tasks. *Inf Process Manag* 2009;45(4):427–37.
8. Dinov ID. Data science and predictive analytics: biomedical and health applications using R. Cham: Springer; 2018.
9. Simon GJ, Aliferis C, editors. Artificial intelligence and machine learning in health care and medical sciences: best practices and pitfalls. *Springer* 2024.
10. Murphy KP. Machine learning: a probabilistic perspective. Cambridge: MIT Press; 2012.
11. Powers DMW. Evaluation: from precision, recall and F-measure to ROC, informedness, markedness and correlation. *Int J Mach Learn Technol* 2011;2(1):37–63.
