# Supplementary material for: Beyond Area Under the Receiver Operating Characteristic Curve: Evaluating Predictive Performance Metrics Under Class Imbalance in Real-World Clinical Data
Source: JMIR Form Res. 2026 Jun 24;10:e86379. doi: 10.2196/86379 (PMC13293568; doi:10.2196/86379)
Supplement: Multimedia Appendix 8 [file formative-v10-e86379-s008.docx]

**Multimedia Appendix 8. Checklist for updated quality, risk of bias, and applicability assessment tool for prediction models using regression or artificial intelligence methods (PROBAST+AI).**

| **Domain and Item** | **Checklist item** | **Development** | **Internal Evaluation** |
| --- | --- | --- | --- |
| **Participants** |  |  |  |
| 1.1 | Were appropriate data sources used? | Yes (hospital cohort of COVID-19 patients, aligned with the clinical scenario for prognostic prediction) | Yes (hospital cohort of COVID-19 patients, aligned with the clinical scenario for prognostic prediction) |
| 1.2 | Was an appropriate study design used? | Yes (a cohort design has been used) | Yes (a cohort design has been used) |
| 1.3 | Did the in- and exclusions of study participants result in a representative data set? | Yes (broad inclusion criteria, no artificial exclusions, and a sample covering a wide range of clinical severity) | Yes (broad inclusion criteria, no artificial exclusions, and a sample covering a wide range of clinical severity) |
| Risk of bias introduced by participants or data sources | | low risk of bias | low risk of bias |
| Applicability Assessment | | low concern | low concern |
| **Predictors** |  |  |  |
| 2.1 | Were predictors defined and assessed in a similar way for all participants? | Yes (definitions of predictors and their assessment were similar for all participants) | Yes (definitions of predictors and their assessment were similar for all participants) |
| 2.2 | Was any pre-processing of predictors similar for all participants? | Yes (predictors were used as recorded, without additional preprocessing) | Yes (predictors were used as recorded, without additional preprocessing) |
| 2.3 | Were predictor assessments made without knowledge of outcome data? | Yes (predictors were recorded before the outcome and extracted independently of outcome information) | Yes (predictors were recorded before the outcome and extracted independently of outcome information) |
| 2.4 | Were the predictors included in the model available at the time the model was intended to be used? | Yes (predictors were available at the intended time of model use) | Yes (predictors were available at the intended time of model use) |
| Risk of bias introduced by predictors or their assessment | | low risk of bias | low risk of bias |
| Applicability Assessment |  | low concern | low concern |
| **Outcome** |  |  |  |
| 3.1 | Were outcomes defined and assessed appropriately? | Yes (objective outcomes were used) | Yes (objective outcomes were used) |
| 3.2 | Were outcomes defined and assessed in a similar way for all participants? | Yes (outcomes were defined and determined in a similar way for all participants) | Yes (outcomes were defined and determined in a similar way for all participants) |
| 3.3 | Were outcome assessments made without use or knowledge of predictor data? | Yes (outcomes were ascertained from clinical records independently of model predictors) | Yes (outcomes were ascertained from clinical records independently of model predictors) |
| 3.4 | Was the time interval between predictor assessment and outcome assessment appropriate? | Yes (predictors were measured before the outcome and at a clinically appropriate time for prediction) | Yes (predictors were measured before the outcome and at a clinically appropriate time for prediction) |
| Risk of bias introduced by outcomes or their assessment | | low risk of bias | low risk of bias |
| Applicability Assessment |  | low concern | low concern |
| **Analysis** |  |  |  |
| 4.1 | Was there evidence that the sample size was reasonable? | Yes (the sample size and number of events were adequate) | Yes (the sample size and number of events were adequate) |
| 4.2 | Were continuous and categorical predictors handled appropriately? | Yes (continuous variables were handled as continuous, and categorical predictors were encoded using standard methods) | NA |
| 4.3 | Were participants with missing or censored data handled appropriately in the analysis? | Yes (missing data were handled internally by the machine learning algorithm, without imputation or outcome-based exclusion) | Yes (missing data were handled internally by the machine learning algorithm, without imputation or outcome-based exclusion) |
| 4.4 | If methods to address class imbalance were used, was the model or the model predictions recalibrated? | No recalibration was applied; calibration and per-class metrics were evaluated were assessed on the original and resampled datasets | No recalibration was applied; calibration and per-class metrics were evaluated were assessed on the original and resampled datasets |
| 4.5 | Were methods used to address potential model overfitting? | Yes (10-fold cross-validation have been used to address potential overfitting) | NA |
| 4.6 | Was model evaluation based on only apparent performance avoided? | NA | Yes (cross-validation was implemented to avoid reliance on apparent performance) |
| 4.10 | If data splitting was done to create training and test datasets, was there evidence that data leakage was avoided? | Yes (cross-validation was implemented to avoid data leakage between training and test data) | Yes (cross-validation was implemented to avoid data leakage between training and test data) |
| 4.11 | If resampling methods were used to evaluate model performance, were all model development steps replicated in the resampling process? | Yes (all model development steps were replicated in the resampling process) | Yes (all model development steps were replicated in the resampling process) |
| 4.12 | Was the predictive performance of the model evaluated appropriately, e.g., calibration, discrimination, and net benefit? | Yes (multiple performance complementary metrics, calibration and net benefit were used) | Yes (multiple performance complementary metrics, calibration and net benefit were used) |
| Risk of bias introduced by the analysis | | low risk of bias | low risk of bias |

NA: Not applicable.
