# Supplementary figures and images for: Beyond Area Under the Receiver Operating Characteristic Curve: Evaluating Predictive Performance Metrics Under Class Imbalance in Real-World Clinical Data
Source: JMIR Form Res. 2026 Jun 24;10:e86379. doi: 10.2196/86379 (PMC13293568; doi:10.2196/86379)

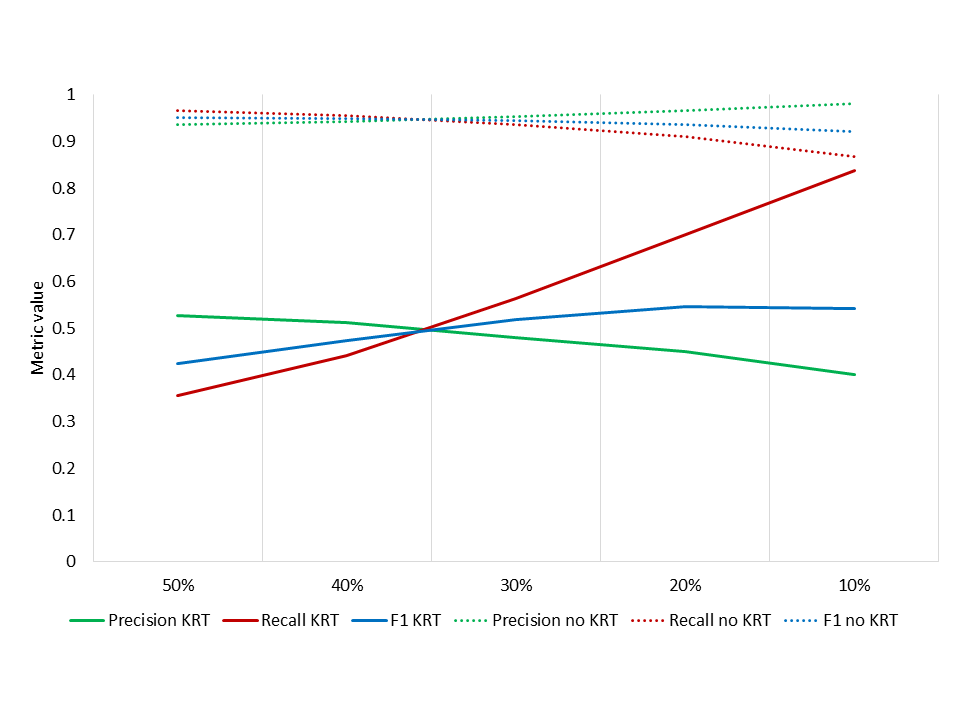

Supplement: Multimedia Appendix 9 [file formative-v10-e86379-s009.png]

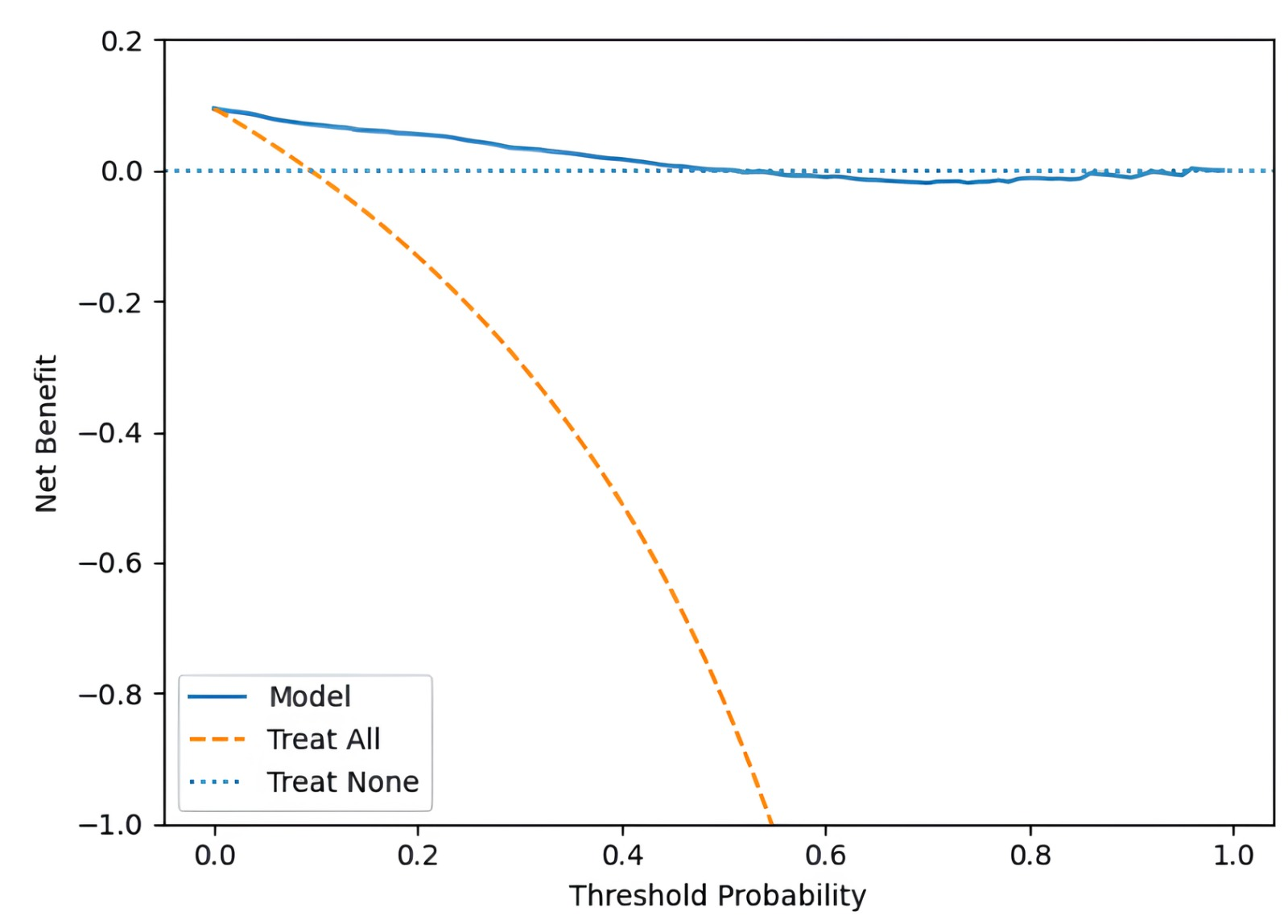

Supplement: Multimedia Appendix 10 [file formative-v10-e86379-s010.png]

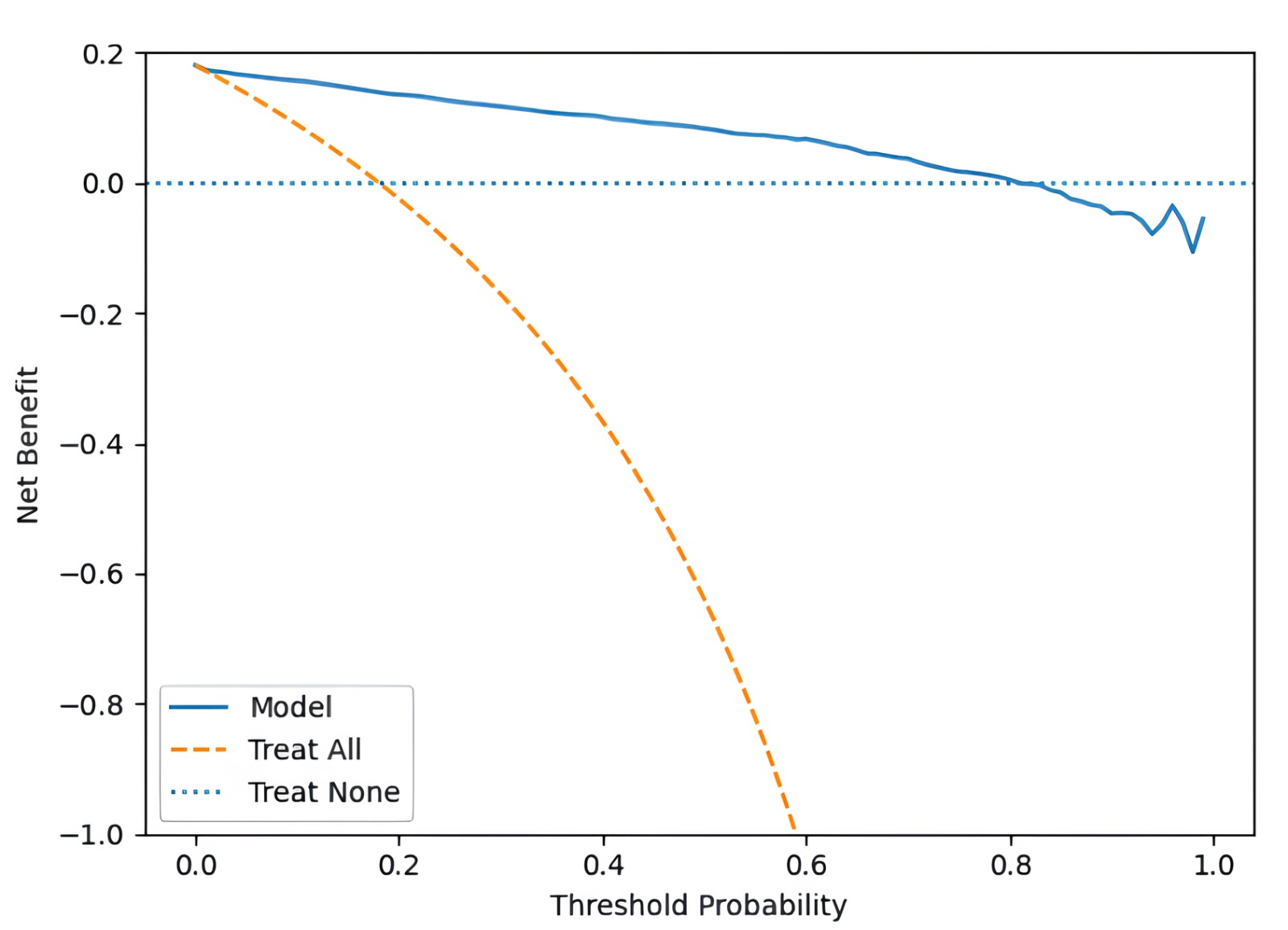

Supplement: Multimedia Appendix 11 [file formative-v10-e86379-s011.png]

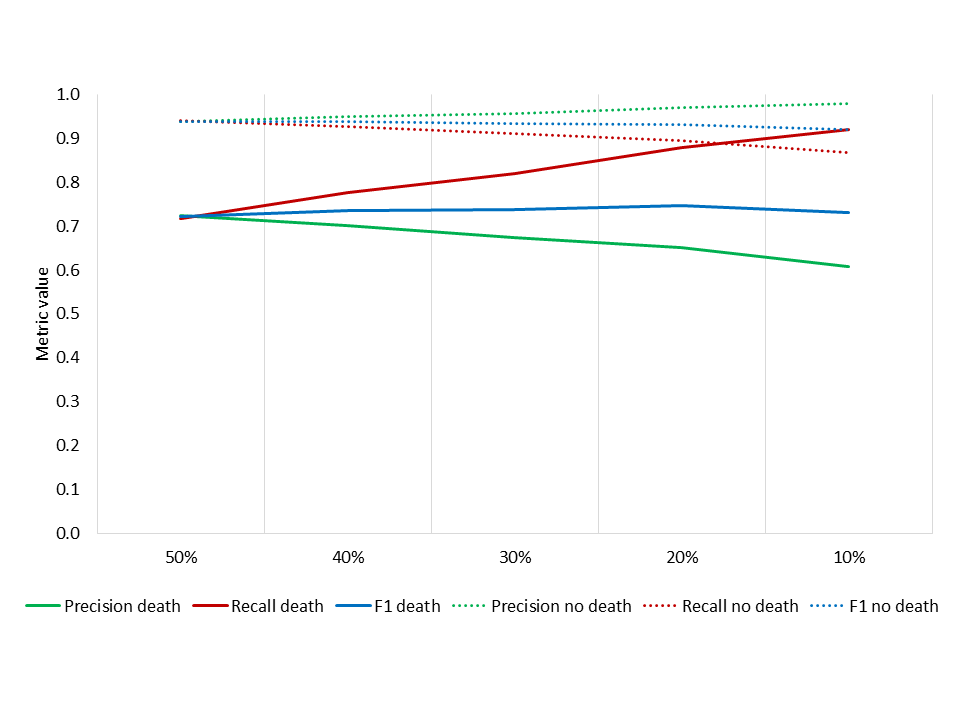

Supplement: Multimedia Appendix 16 [file formative-v10-e86379-s016.png]
