# Supplementary material for: Beyond Area Under the Receiver Operating Characteristic Curve: Evaluating Predictive Performance Metrics Under Class Imbalance in Real-World Clinical Data
Source: JMIR Form Res. 2026 Jun 24;10:e86379. doi: 10.2196/86379 (PMC13293568; doi:10.2196/86379)
Supplement: Multimedia Appendix 12 [file formative-v10-e86379-s012.docx]

| **Multimedia Appendix 12: Global and per-class metrics for different rebalancing techniques for kidney replacement therapy**. | | | | | | | | | | | |
| --- | --- | --- | --- | --- | --- | --- | --- | --- | --- | --- | --- |
| **Method** | **Accuracy** | **AUROC** | **Macro-F1** | **KRT CLASS** | | | **NO KRT CLASS** | | | **Brier** | **TPRGap** |
|  |  |  |  | **F1** | **Precision** | **Recall** | **F1** | **Precision** | **Recall** |  |  |
| **Unbalanced** | 0.910  (0.906-0.914) | 0.928  (0.923-0.933) | 0.695  (0.682-0.708) | 0.439  (0.414-0.436) | 0.539  (0.508-0.580) | 0.372  (0.345-0.399) | 0.951  (0.949-0.953) | 0.936  (0.934-0.938) | 0.966  (0.961-0.971) | 0.066  (0.063-0.070) | 0.597  (0.570-0.625) |
| **RUS** | 0.833  (0.828-0.839) | 0.924  (0.920-0.929) | 0.710  (0.703-0.716) | 0.521  (0.511-0.530) | 0.358  (0.350-0.366) | 0.952  (0.942-0.962) | 0.899  (0.896-0.903) | 0.994  (0.993-0.995) | 0.821  (0.816-0.826) | 0.141  (0.137-0.145) | 0.131  (0.122-0.140) |
| **UBR** | 0.892  (0.889-0.895) | 0.926  (0.921-0.931) | 0.733  (0.726-0.740) | 0.527  (0.514-0.539) | 0.450  (0.440-0.461) | 0.634  (0.615-0.653) | 0.939  (0.937-0.941) | 0.960  (0.958-0.962) | 0.919  (0.916-0.921) | 0.075  (0.073-0.077) | 0.285  (0.266-0.304) |
| **e2sc_us** | 0.889  (0.884-0.893) | 0.925  (0.920-0.929) | 0.725  (0.718-0.733) | 0.514  (0.500-0.527) | 0.440  (0.425-0.456) | 0.618  (0.598-0.638) | 0.937  (0.934-0.940) | 0.958  (0.956-0.960) | 0.917  (0.912-0.923) | 0.076  (0.073-0.078) | 0.299  (0.277-0.322) |
| **CNN** | 0.869  (0.863-0.876) | 0.923  (0.917-0.929) | 0.726  (0.716-0.736) | 0.528  (0.512-0.544) | 0.403  (0.388-0.417) | 0.768  (0.747-0.789) | 0.924  (0.920-0.928) | 0.973  (0.971-0.975) | 0.880  (0.874-0.886) | 0.092  (0.088-0.096) | 0.112  (0.091-0.133) |
| **NM1** | 0.770  (0.759-0.781) | 0.895  (0.888-0.902) | 0.646  (0.636-0.656) | 0.436  (0.424-0.449) | 0.285  (0.274-0.295) | 0.934  (0.925-0.943) | 0.855  (0.847-0.864) | 0.991  (0.990-0.992) | 0.753  (0.740-0.765) | 0.199  (0.190-0.208) | 0.181  (0.165-0.197) |
| **NM2** | 0.235  (0.224-0.247) | 0.816  (0.808-0.824) | 0.233  (0.223-0.243) | 0.190  (0.187-0.193) | 0.106  (0.104-0.107) | 0.942  (0.934-0.950) | 0.276  (0.258-0.294) | 0.963  (0.957-0.969) | 0.161  (0.149-0.173) | 0.752  (0.740-0.763) | 0.781  (0.768-0.794) |
| **ROS** | 0.893  (0.889-0.896) | 0.926  (0.922-0.931) | 0.728  (0.719-0.737) | 0.516  (0.500-0.532) | 0.452  (0.438-0.466) | 0.602  (0.577-0.626) | 0.940  (0.938-0.942) | 0.957  (0.954-0.959) | 0.923  (0.920-0.927) | 0.073  (0.071-0.076) | 0.322  (0.297-0.346) |
| **ADASYN** | 0.906  (0.901-0.911) | 0.926  (0.921-0.932) | 0.702  (0.688-0.716) | 0.455  (0.429-0.481) | 0.510  (0.476-0.543) | 0.413  (0.388-0.439) | 0.949  (0.946-0.952) | 0.940  (0.937-0.942) | 0.958  (0.953-0.963) | 0.065  (0.062-0.067) | 0.545  (0.519-0.570) |
| **SMOTE** | 0.906  (0.902-0.910) | 0.928  (0.923-0.932) | 0.704  (0.693-0.715) | 0.459  (0.439-0.480) | 0.508  (0.482-0.533) | 0.420  (0.399-0.440) | 0.949  (0.946-0.951) | 0.940  (0.938-0.942) | 0.957  (0.953-0.961) | 0.064  (0.062-0.066) | 0.537  (0.517-0.557) |
| **BorderlineSMOTE** | 0.906  (0.902-0.910) | 0.926  (0.921-0.932) | 0.703  (0.694-0.713) | 0.458  (0.440-0.476) | 0.511  (0.483-0.538) | 0.417  (0.395-0.440) | 0.949  (0.946-0.951) | 0.940  (0.938-0.942) | 0.957  (0.953-0.962) | 0.065  (0.063-0.068) | 0.540  (0.515-0.565) |
| **SVMSMOTE** | 0.906  (0.902-0.910) | 0.925  (0.920-0.931) | 0.701  (0.689-0.713) | 0.453  (0.431-0.476) | 0.506  (0.483-0.530) | 0.412  (0.384-0.440) | 0.948  (0.946-0.951) | 0.939  (0.937-0.942) | 0.958  (0.954-0.962) | 0.066  (0.063-0.068) | 0.546  (0.516-0.575) |
| **KMeansSMOTE** | 0.905  (0.902-0.908) | 0.926  (0.921-0.930) | 0.691  (0.681-0.701) | 0.434  (0.415-0.453) | 0.502  (0.483-0.521) | 0.383  (0.360-0.407) | 0.948  (0.947-0.950) | 0.937  (0.935-0.939) | 0.960  (0.957-0.963) | 0.066  (0.064-0.068) | 0.577  (0.551-0.602) |

The data are presented as the mean and 95% confidence interval.

ADASYN: adaptive synthetic, AUROC: area under the receiver operating characteristic curve, BorderlineSMOTE: borderline synthetic minority oversampling technique, CNN: condensed nearest neighbour, e2sc_us: effective, efficient, and scalable confidence-based undersampling, KMeansSMOTE: K-means synthetic minority oversampling technique, KRT: kidney replacement therapy, NM1: near miss 1, NM2: near miss 2, ROS: random oversampling, RUS: random undersampling, SMOTE: the synthetic minority over-sampling technique, SVMSMOTE: support vector machine synthetic minority oversampling technique, TL: Tomek links, UBR: redundancy-based undersampling.
