# Supplementary material for: Beyond Area Under the Receiver Operating Characteristic Curve: Evaluating Predictive Performance Metrics Under Class Imbalance in Real-World Clinical Data
Source: JMIR Form Res. 2026 Jun 24;10:e86379. doi: 10.2196/86379 (PMC13293568; doi:10.2196/86379)
Supplement: Multimedia Appendix 13 [file formative-v10-e86379-s013.docx]

| **Multimedia Appendix 13: Global and per-class metrics for different rebalancing techniques for death.** | | | | | | | | | | | |
| --- | --- | --- | --- | --- | --- | --- | --- | --- | --- | --- | --- |
| **Method** | **Accuracy** | **AUROC** | **Macro-F1** | **DEATH CLASS** | | | **NO DEATH CLASS** | | | **Brier** | **TPRGap** |
|  |  |  |  | **F1** | **Precision** | **Recall** | **F1** | **Precision** | **Recall** |  |  |
| **Unbalanced** | 0.900  (0.896-0.903) | 0.945  (0.939-0.95) | 0.830  (0.825-0.835) | 0.721  (0.712-0.729) | 0.725  (0.703-0.747) | 0.718  (0.71-0.725) | 0.939  (0.937-0.941) | 0.938  (0.934-0.941) | 0.940  (0.934-0.946) | 0.072  (0.069-0.075) | 0.230  (0.217-0.242) |
| **RUS** | 0.880  (0.874-0.886) | 0.94  (0.938-0.947) | 0.828  (0.820-0.836) | 0.733  (0.720-0.746) | 0.612  (0.595-0.629) | 0.917  (0.910-0.924) | 0.922  (0.918-0.927) | 0.979  (0.977-0.982) | 0.872  (0.865-0.878) | 0.101  (0.095-0.106) | 0.045  (0.038-0.053) |
| **UBR** | 0.890  (0.884-0.896) | 0.941  (0.937-0.946) | 0.834  (0.826-0.843) | 0.738  (0.725-0.751) | 0.648  (0.627-0.668) | 0.859  (0.847-0.872) | 0.930  (0.927-0.934) | 0.966  (0.963-0.970) | 0.897  (0.890-0.904) | 0.084  (0.079-0.089) | 0.043  (0.034-0.051) |
| **e2sc_us** | 0.889  (0.884-0.894) | 0.941  (0.936-0.945) | 0.833  (0.825-0.841) | 0.736  (0.723-0.749) | 0.646  (0.627-0.664) | 0.857  (0.848-0.866) | 0.930  (0.927-0.933) | 0.966  (0.963-0.969) | 0.896  (0.890-0.902) | 0.084  (0.079-0.088) | 0.039  (0.029-0.050) |
| **CNN** | 0.886  (0.880-0.891) | 0.939  (0.934-0.944) | 0.830  (0.823-0.838) | 0.734  (0.722-0.745) | 0.634  (0.617-0.651) | 0.872  (0.859-0.884) | 0.927  (0.923-0.931) | 0.969  (0.965-0.973) | 0.889  (0.883-0.896) | 0.088  (0.084-0.092) | 0.025  (0.017-0.033) |
| **NM1** | 0.710  (0.701-0.719) | 0.882  (0.874-0.890) | 0.663  (0.654-0.671) | 0.536  (0.524-0.548) | 0.377  (0.366-0.388) | 0.928  (0.922-0.934) | 0.789  (0.782-0.797) | 0.977  (0.975-0.979) | 0.662  (0.652-0.672) | 0.238  (0.231-0.246) | 0.266  (0.257-0.275) |
| **NM2** | 0.357  (0.350-0.363) | 0.809  (0.800-0.819) | 0.356  (0.350-0.363) | 0.345  (0.337-0.353) | 0.211  (0.205-0.217) | 0.939  (0.933-0.945) | 0.368  (0.359-0.376) | 0.944  (0.937-0.951) | 0.228  (0.222-0.235) | 0.633  (0.627-0.638) | 0.711  (0.703-0.719) |
| **ROS** | 0.894  (0.889-0.899) | 0.941  (0.936-0.945) | 0.834  (0.826-0.841) | 0.733  (0.721-0.745) | 0.673  (0.654-0.692) | 0.807  (0.793-0.821) | 0.934  (0.931-0.937) | 0.955  (0.951-0.959) | 0.914  (0.908-0.919) | 0.078  (0.075-0.081) | 0.107  (0.092-0.122) |
| **ADASYN** | 0.898  (0.894-0.902) | 0.942  (0.937-0.947) | 0.830  (0.824-0.835) | 0.722  (0.712-0.731) | 0.711  (0.701-0.721) | 0.734  (0.718-0.749) | 0.938  (0.935-0.940) | 0.941  (0.936-0.945) | 0.934  (0.932-0.937) | 0.073  (0.070-0.076) | 0.200  (0.184-0.217) |
| **SMOTE** | 0.898  (0.894-0.901) | 0.942  (0.937-0.947) | 0.829  (0.823-0.835) | 0.721  (0.711-0.731) | 0.709  (0.697-0.721) | 0.733  (0.716-0.751) | 0.937  (0.935-0.940) | 0.941  (0.936-0.945) | 0.934  (0.931-0.937) | 0.074  (0.071-0.076) | 0.200  (0.182-0.219) |
| **BorderlineSMOTE** | 0.898  (0.894-0.902) | 0.941  (0.936-0.946) | 0.829  (0.824-0.835) | 0.721  (0.712-0.730) | 0.713  (0.695-0.731) | 0.730  (0.720-0.741) | 0.938  (0.935-0.940) | 0.940  (0.937-0.944) | 0.935  (0.931-0.940) | 0.074  (0.071-0.077) | 0.205  (0.192-0.218) |
| **SVMSMOTE** | 0.899  (0.896-0.903) | 0.943  (0.939-0.948) | 0.832  (0.827-0.837) | 0.726  (0.718-0.734) | 0.713  (0.698-0.728) | 0.741  (0.731-0.750) | 0.938  (0.936-0.941) | 0.942  (0.939-0.946) | 0.934  (0.930-0.938) | 0.072  (0.070-0.075) | 0.194  (0.183-0.204) |
| **KMeansSMOTE** | 0.898  (0.893-0.903) | 0.941  (0.935-0.946) | 0.829  (0.822-0.835) | 0.720  (0.709-0.730) | 0.716  (0.699-0.734) | 0.724  (0.711-0.736) | 0.938  (0.935-0.941) | 0.939  (0.935-0.943) | 0.937  (0.932-0.942) | 0.074  (0.071-0.078) | 0.213  (0.199-0.227) |

ADASYN: adaptive synthetic, AUROC: area under the receiver operating characteristic curve, BorderlineSMOTE: borderline synthetic minority oversampling technique, CNN: condensed nearest neighbour, e2sc_us: effective, efficient, and scalable confidence-based undersampling, KMeansSMOTE: K-means synthetic minority oversampling technique, NM1: near miss 1, NM2: near miss 2, ROS: random oversampling, RUS: random undersampling, SMOTE: the synthetic minority over-sampling technique, SVMSMOTE: support vector machine synthetic minority oversampling technique, TL: Tomek links, UBR: redundancy-based undersampling.
