# Supplementary material for: Beyond Area Under the Receiver Operating Characteristic Curve: Evaluating Predictive Performance Metrics Under Class Imbalance in Real-World Clinical Data
Source: JMIR Form Res. 2026 Jun 24;10:e86379. doi: 10.2196/86379 (PMC13293568; doi:10.2196/86379)
Supplement: Multimedia Appendix 14 [file formative-v10-e86379-s014.docx]

Multimedia Appendix 14. Outcomes and evaluation metrics of predictive scores for COVID-19 patients based on the DynaMed summary.

| **Study** | **Outcome** | **Evaluation metrics** |
| --- | --- | --- |
| Amezcua-Guerra LM et al., 2021 [1]; González-Flores J et al., 2021 [2] | Thrombosis, IMV, mortality | AUROC, PPV, NPV, positive and negative likelihood ratio |
| Bello-Chavolla OY et al., 2020 [3] | Mortality | AUROC |
| Berenguer J et al., 2021 [4] | Mortality | AUROC, plot of the expected and observed result |
| Chua F et al., 2021 [5] | Mortality | AUROC, plot of the expected and observed result |
| Çınar T et al., 2021 [6] | Mortality | AUROC |
| Figueiredo FA et al., 2022 [7]; Ventura VGJ et al., 2023 [8] | KRT | AUROC, plot of the expected and observed result, Brier score |
| García-Ortega A et al., 2021 [9] | Embolism | AUROC |
| Gavin W et al., 2021 [10] | Readmission | Brier score |
| Giamarellos-Bourboulis EJ et al., 2022 [11] | Respiratory failure | AUROC, sensitivity, specificity, PPV, NPV |
| Haimovich AD et al., 2020 [12] | Severe illness | AUROC, accuracy, sensitivity, specificity, AUPRC, F1 score, balanced accuracy, Brier score |
| Hajifathalian K et al., 2020 [13] | Mortality | AUROC, plot of the expected and observed result |
| Hwang J et al., 2021 [14] | Mortality | AUROC, Hosmer-Lemeshow test |
| Jamal MH et al., 2020 [15] | Severe illness | AUROC |
| Ji D et al., 2020  Erturk Sengel B et al., 2021 [16] | Severe illness | AUROC, PPV, NPV |
| Karaaslan T et al., 2022 [17] | Mortality | AUROC |
| Knight SR et al., 2020 [18]; Aletreby WT et al., 2022 [19] | Mortality | AUROC, Brier score |
| Kurt E et al., 2022 [20]; Ak R et al., 2021 [21] | In-hospital mortality and critical care requirement | AUROC |
| Liang W et al., 2020 [22] | Severe illness | AUROC |
| Liu H et al., 2021 [23] | Mortality | AUROC, accuracy, sensitivity, specificity, PPV, NPV |
| Marcolino MS et al., 2021 [24] | Mortality | AUROC, plot of the expected and observed result, Brier score |
| Martínez-Lacalzada M et al., 2021 [25] | Severe illness | AUROC, plot of the expected and observed result, Brier score |
| Smith DS et al., 2020 [26] | Diagnosis of COVID-19 | AUROC, plot of the expected and observed result |
| Valente Silva B et al., 2022 [27] | Mortality | AUROC |
| van Dam PM et al., 2021 [28] | Mortality, ICU admission plus mortality | AUROC, plot of the expected and observed result, Brier score, sensitivity, specificity, PPV, NPV |
| Yan L et al., 2020 [29] | Mortality | AUROC, accuracy, precision, recall, F1 score, TP, FP, TN, FN, of the entire set and by class |

AUPRC: area under the precision/recall curve; AUROC: area under the receiving operator characteristic curve; ICU: intensive care unit; IMV: Invasive Mechanical Ventilation, KRT: kidney replacement therapy; NPV: negative predictive value; PPV: positive predictive value; TN: true negative; TP: true positive.

# References

1. Amezcua-Guerra LM, Audelo K, Guzmán J, et al. A simple and readily available inflammation-based risk scoring system on admission predicts the need for mechanical ventilation in patients with COVID-19. *Inflamm Res* 2021;70:731–42.
2. González-Flores J, García-Ávila C, Springall R, et al. Usefulness of Easy-to-Use Risk Scoring Systems Rated in the Emergency Department to Predict Major Adverse Outcomes in Hospitalized COVID-19 Patients. *J Clin Med* 2021;10:3657.
3. Bello-Chavolla OY, et al. Predicting Mortality Due to SARS-CoV-2: A Mechanistic Score Relating Obesity and Diabetes to COVID-19 Outcomes in Mexico. *J Clin Endocrinol Metab* 2020;105:dgaa346.
4. Berenguer J, Bahena-López JP, Antonio-Villa NE, et al. Development and validation of a prediction model for 30-day mortality in hospitalised patients with COVID-19: the COVID-19 SEIMC score. *Thorax* 2021;76:920–9.
5. Chua F, Vancheeswaran R, Draper A, et al. Early prognostication of COVID-19 to guide hospitalisation versus outpatient monitoring using a point-of-test risk prediction score. *Thorax* 2021;76:696–703.
6. Çınar T, Hayıroğlu Mİ, Çiçek V, et al. Is prognostic nutritional index a predictive marker for estimating all-cause in-hospital mortality in COVID-19 patients with cardiovascular risk factors? *Heart Lung* 2021;50:307–12.
7. Figueiredo FA, Ramos LEF, Silva RT, et al. Development and validation of the MMCD score to predict kidney replacement therapy in COVID-19 patients. *BMC Med* 2022;20:324.
8. Ventura VGJ, Delfino-Pereira P, Pires MC, et al. Temporal validation of the MMCD score to predict kidney replacement therapy and in-hospital mortality in COVID-19 patients. BMC *Nephrol* 2023;24:292.
9. García-Ortega A, Oscullo G, Calvillo P, et al. Incidence, risk factors, and thrombotic load of pulmonary embolism in patients hospitalized for COVID-19 infection. *J Infect* 2021;82:261–9.
10. Gavin W, Rager J, Russ J, et al. Accuracy of the Simplified HOSPITAL Score in Predicting COVID-19 Readmissions—Exploring Outcomes from a Hospital-at-Home Program. *J Healthc Manag* 2021;67:54–62.
11. Giamarellos-Bourboulis EJ, Poulakou G, Nooijer A, et al. Development and validation of SCOPE score: A clinical score to predict COVID-19 pneumonia progression to severe respiratory failure. *Cell Rep Med* 2022;3:100560.
12. Haimovich AD, Ravindra NG, Stoytchev S, et al. Development and Validation of the Quick COVID-19 Severity Index: A Prognostic Tool for Early Clinical Decompensation. *Ann Emerg Med* 2020;76:442–53.
13. Hajifathalian K, Sharaiha RZ, Kumar S, et al. Development and external validation of a prediction risk model for short-term mortality among hospitalized U.S. COVID-19 patients: A proposal for the COVID-AID risk tool. *PLoS One* 2020;15:e0239536.
14. Hwang J, Park SH, Lee SW, et al. Predictors of mortality in thrombotic thrombocytopenia after adenoviral COVID-19 vaccination: the FAPIC score. *Eur Heart J* 2021;42:4053–63.
15. Jamal MH, et al. A biomarker based severity progression indicator for COVID-19: the Kuwait prognosis indicator score. *Biomarkers* 2020;25:641–8.
16. Ji D, Doi SA, AlYouha S, et al. Prediction for Progression Risk in Patients With COVID-19 Pneumonia: The CALL Score. *Clin Infect Dis* 2020;71:1393–9.
17. Karaaslan T, Karaaslan E. Predictive Value of Systemic Immune-inflammation Index in Determining Mortality in COVID-19 Patients. *J Crit Care Med* 2022;8:156–64.
18. Knight SR, Ho A, Pius R, et al. Risk stratification of patients admitted to hospital with covid-19 using the ISARIC WHO Clinical Characterisation Protocol: development and validation of the 4C Mortality Score. *BMJ* 2020;370:m3339.
19. Aletreby WT, Mumtaz SA, Shahzad SA, et al. External Validation of 4C ISARIC Mortality Score in Critically ill COVID-19 Patients from Saudi Arabia. Saudi J Med Med Sci 2022;10:19–24.
20. Kurt E, Bahadirli S. The Usefulness of Shock Index and Modified Shock Index in Predicting the Outcome of COVID-19 Patients. *Disaster Med Public Health Prep* 2021;16:1–6.
21. Ak R, Doğanay F. Comparison of 4 Different Threshold Values of Shock Index in Predicting Mortality of COVID-19 Patients. *Disaster Med Public Health Prep* 2021;17:e99.
22. Liang W, Liang H, Ou L, et al. Development and Validation of a Clinical Risk Score to Predict the Occurrence of Critical Illness in Hospitalized Patients With COVID-19. *JAMA Intern Med* 2020;180:1081–9.
23. Liu H, Chen J, Yang Q, et al. Development and validation of a risk score using complete blood count to predict in-hospital mortality in COVID-19 patients. *Med* 2021;2:435–47.e4.
24. Marcolino MS, Pires MC, Ramos LEF, et al. ABC2-SPH risk score for in-hospital mortality in COVID-19 patients: development, external validation and comparison with other available scores. *Int J Infect Dis* 2021;110:281–308.
25. Martínez-Lacalzada M, Viteri-Noël A, Manzano L, et al. Predicting critical illness on initial diagnosis of COVID-19 based on easily obtained clinical variables: development and validation of the PRIORITY model. *Clin Microbiol Infect* 2021;27:1838–44.
26. Smith DS, Richey EA, Brunetto WL. A Symptom-Based Rule for Diagnosis of COVID-19. *SN Compr Clin Med* 2020;2:1947–54.
27. Silva BV, Plácido R, Jorge C, et al. Prognostic Accuracy of the Modified CHA2DS2-VASc Score in COVID-19 Patients Admitted to the Emergency Department Due to Clinical Worsening. *Acta Med Port* 2021;34:433–42.
28. Dam PM van, Zelis N, Stassen P, et al. Validating the RISE UP score for predicting prognosis in patients with COVID-19 in the emergency department: a retrospective study. *BMJ Open* 2021;11:e045141.
29. Yan L, Zhang H-T, Goncalves J, et al. An interpretable mortality prediction model for COVID-19 patients. *Nat Mach Intell* 2020;2:283–8.
