# Supplementary material for: Beyond Area Under the Receiver Operating Characteristic Curve: Evaluating Predictive Performance Metrics Under Class Imbalance in Real-World Clinical Data
Source: JMIR Form Res. 2026 Jun 24;10:e86379. doi: 10.2196/86379 (PMC13293568; doi:10.2196/86379)
Supplement: Multimedia Appendix 15 [file formative-v10-e86379-s015.docx]

Multimedia Appendix 15. Outcomes and evaluation metrics of predictive scores for cardiovascular disease based on the DynaMed summary.

| **Study** | **Outcome** | **Evaluation metrics** |
| --- | --- | --- |
| Assmann G et al., 2002 [1] | 10-year risk of acute coronary events | AUROC, Greenwood–Nam–D’Agostino test |
| Ridker PM et al., 2007 [2] | 10-year cardiovascular disease risk in women | C statistic, Hosmer-Lemeshow statistic, Brier score |
| Ridker PM et al., 2008 [3] | 10-year cardiovascular disease risk in men | C statistic, Hosmer-Lemeshow statistic |
| D'Agostino RB Sr et al., 2008 [4] | 10-year risk of cardiovascular events in adults | C statistic, Hosmer-Lemeshow statistic, sensitivity and specificity of the top quintile of predicted risk |
| Cederholm J et al., 2011 [5] | 5-year risk of cardiovascular disease in type 1 diabetes | C statistic, Hosmer-Lemeshow statistic, sensitivity and specificity of the top quartile of predicted risk |
| Goff DC Jr et al., 2014 [6] | 10-year risk of coronary heart disease | C statistic, calibration chi-square statistics |
| McClelland RL et al., 2015 [7] | 10-year risk of coronary heart disease | C statistic, calibration slope and calibration-in-the-large |
| Yadlowsky S et al., 2018 [8] | 10-year risk of myocardial infarction, death from coronary heart disease, or stroke | C statistic, calibration slope |
| Khan SS et al., 2024 [9] | Atherosclerotic cardiovascular disease and heart failure | C statistic, calibration curve predicted values versus observed values and calculation of the curve slope |

**References**

1. Assmann G, Cullen P, Schulte H. Simple scoring scheme for calculating the risk of acute coronary events based on the 10-year follow-up of the prospective cardiovascular Münster (PROCAM) study. *Circulation* 2002 Jan 22;105(3):310-5.
2. Ridker PM, Buring JE, Rifai N, et al. Development and validation of improved algorithms for the assessment of global cardiovascular risk in women: the Reynolds Risk Score. *JAMA* 2007;297(6):611-9.
3. Ridker PM, Paynter NP, Rifai N, et al. C-reactive protein and parental history improve global cardiovascular risk prediction: the Reynolds Risk Score for men. *Circulation* 2008;118(22):2243-51, 4p following 2251.
4. D'Agostino RB Sr, Vasan RS, Pencina MJ, et al. General cardiovascular risk profile for use in primary care: the Framingham Heart Study. *Circulation* 2008 Feb 12;117(6):743-53.
5. Cederholm J, Eeg-Olofsson K, Eliasson B, et al. A new model for 5-year risk of cardiovascular disease in Type 1 diabetes; from the Swedish National Diabetes Register (NDR). *Diabet Med* 2011;28(10):1213-20.
6. Goff DC Jr, Lloyd-Jones DM, Bennett G, et al. 2013 ACC/AHA guideline on the assessment of cardiovascular risk: a report of the American College of Cardiology/American Heart Association Task Force on Practice Guidelines. *Circulation* 2014;129(25 Suppl 2):S49-73.
7. McClelland RL, Jorgensen NW, Budoff M, et al. 10-Year Coronary Heart Disease Risk Prediction Using Coronary Artery Calcium and Traditional Risk Factors: Derivation in the MESA (Multi-Ethnic Study of Atherosclerosis) With Validation in the HNR (Heinz Nixdorf Recall) Study and the DHS (Dallas Heart Study). *J Am Coll Cardiol* 2015;66(15):1643-53.
8. Yadlowsky S, Hayward RA, Sussman JB, et al. Clinical Implications of Revised Pooled Cohort Equations for Estimating Atherosclerotic Cardiovascular Disease Risk. *Ann Intern Med* 2018;169(1):20-29.
9. Khan SS, Matsushita K, Sang Y, et al. Development and Validation of the American Heart Association's PREVENT Equations. *Circulation* 2024;149(6):430-449.
