# Supplementary material for: Beyond Area Under the Receiver Operating Characteristic Curve: Evaluating Predictive Performance Metrics Under Class Imbalance in Real-World Clinical Data
Source: JMIR Form Res. 2026 Jun 24;10:e86379. doi: 10.2196/86379 (PMC13293568; doi:10.2196/86379)
Supplement: Multimedia Appendix 17 [file formative-v10-e86379-s017.docx]

Multimedia Appendix 17. Features' importance and contribution to the final predictive model kidney replacement therapy.

| **Feature** | **Importance** |
| --- | --- |
| Invasive mechanical ventilation | 0.446 |
| Coronary artery disease | 0.024 |
| Hypertension | 0.021 |
| Rheumatologic disease | 0.021 |
| Creatinine | 0.021 |
| Previous transplantation | 0.019 |
| Vaccine doses | 0.017 |
| Inotrope diastolic | 0.016 |
| Chronic kidney disease | 0.013 |
| Sex | 0.013 |
| Lactate | 0.013 |
| Heart failure | 0.012 |
| Bicarbonate | 0.012 |
| Age | 0.012 |
| Dementia | 0.012 |
| Troponin | 0.011 |
| Smoking | 0.011 |
| Oral anticoagulants | 0.011 |
| Neutrophils | 0.011 |
| Sodium | 0.011 |
