# Supplementary material for: Beyond Area Under the Receiver Operating Characteristic Curve: Evaluating Predictive Performance Metrics Under Class Imbalance in Real-World Clinical Data
Source: JMIR Form Res. 2026 Jun 24;10:e86379. doi: 10.2196/86379 (PMC13293568; doi:10.2196/86379)
Supplement: Multimedia Appendix 18 [file formative-v10-e86379-s018.docx]

Multimedia Appendix 18. Features’ importance and contribution to the final predictive model in-hospital mortality.

| **Feature** | **Importance** |
| --- | --- |
| Invasive mechanical ventilation | 0.487 |
| Previous VTE | 0.035 |
| Age | 0.024 |
| Dementia | 0.022 |
| Ischemic stroke | 0.014 |
| Functional status | 0.014 |
| Urea | 0.013 |
| Immunosuppressant drugs | 0.012 |
| Oral anticoagulants | 0.012 |
| AST | 0.011 |
| Cancer | 0.011 |
| SatO2/FiO2 | 0.011 |
| Creatinine | 0.010 |
| Glasgow come scale | 0.010 |
| Oral anticoagulants | 0.012 |
| HIV infection | 0.010 |
| Bicarbonate | 0.010 |
| Hypertension | 0.010 |
| Platelets | 0.010 |
| Sodium | 0.010 |

SatO2/FiO2: saturation of oxygen / fraction of inspired oxygen ratio; VTE: venous thromboembolism.
